# Microsatellite Characterization of Malaysian Mahseer (*Tor* spp.) for Improvement of Broodstock Management and Utilization

**DOI:** 10.3390/ani11092633

**Published:** 2021-09-08

**Authors:** Poh Chiang Chew, Annie Christianus, Jaapar M. Zudaidy, Md Yasin Ina-Salwany, Chou Min Chong, Soon Guan Tan

**Affiliations:** 1 Institute of Bioscience, Universiti Putra Malaysia, Serdang 43400, Malaysia; chew@dof.gov.my (P.C.C.); salwany@upm.edu.my (M.Y.I.-S.); choumin@upm.edu.my (C.M.C.); 2 Freshwater Fisheries Research Division, Fisheries Research Institute Glami Lemi, Jelebu 71650, Malaysia; zudaidyfrigl@gmail.com; 3 Department of Aquaculture, Faculty of Agriculture, Universiti Putra Malaysia, Serdang 43400, Malaysia; 4 Department of Cell and Molecular Biology, Faculty of Biotechnology and Biomolecular Sciences, Universiti Putra Malaysia, Serdang 43400, Malaysia; wsgtan@gmail.com

**Keywords:** Malaysian mahseer, *Tor tambra*, *Tor tambroides*, microsatellite, genetic diversity, genetic relatedness

## Abstract

**Simple Summary:**

The Malaysian mahseer (*Tor* ssp.) of the family Cyprinidae are indigenous large riverine cyprinids that occur only in Southeast Asia. They are the popular freshwater fish for food, ornamental and recreational fishing. However, their wild populations are now ecologically threatened as their numbers decline drastically over the years due to over-exploitation, natural habitat degradation and water pollution. With successful hatchery production, readily accepted artificial feed and fetched high market value, Malaysian mahseer is now considered a perspective for aquaculture. Stocks were collected from various sources for broodstock development to establish an appropriate base population with desirable characteristics that harbour adequate genetic diversity. Information on the genetic status is essential to formulate appropriate strategies for genetic resources protection and its utilization. Genetic diversity of the broodfish can be assessed rapidly with high precision using polymerase chain reaction (PCR)-based DNA markers.

**Abstract:**

In this study, a mixture of *Tor tambra* and *T. tambroides* with unknown genetic background were collected from 11 localities in Malaysia for broodstock development and sperm cryo-banking. This study aims to assess the microsatellite (simple sequence repeat, SSR) variation, genetic diversity, genetic differentiation, level of gene flow, population structure, genetic relatedness and their demographic aspects among these *Tor* populations, in addition to establishing their SSR profile by employing 22 SSR markers via fragment analysis. Total genomic DNA was extracted from 181 samples (91 cryopreserved milt samples and 90 scale samples of live broodfish). Results showed the *Tor* spp. collection retained their genetic variation but exhibited excessive homozygosity among individuals within population. Moderate genetic differentiation was shown among the populations, with highly significant (*p* < 0.001) fixation indices (F_ST_, F_IS_ and F_IT_). A low gene flow over all loci (Nm 1.548) indicates little genetic variation transfer between populations. The genetic structures of all the populations were successfully resolved into four main clusters by an unweighted pair group method with arithmetic mean (UPGMA) dendrogram generated based on Nei’s genetic distances. The population structures based on principal coordinates analysis (PCoA) and the Bayesian model also suggested four distinct clusters following geographical regions and eight closely related populations. This study provided a useful baseline reference for better genetic management and utilization of the *Tor* spp. stocks in their breeding and conservation programmes.

## 1. Introduction

Malaysian mahseer of the Cyprinidae family is an indigenous fish that occurs only in Southeast Asia [1,2]. Locally, *Tor* mahseer fish are generally known as kelah in Peninsular Malaysia, empurau and semah in Sarawak or pelian in Sabah [3,4,5]. The indigenous *Tor* mahseer comprises two valid species, *Tor tambra* and *T. tambroides* [1,6,7,8]. Confusions always occurred in the identification of these local *Tor* species. As such, their nomenclatures had been revised occasionally for the past few decades [2,9,10]. These ecologically threatened species are classified as data deficient in the IUCN Red List [6,7,11]. Wild populations of Malaysian mahseer are declining due to human activities such as deforestation, uncontrolled logging, agriculture, and over-exploitation that cause natural habitat degradation and water pollution. This is clearly shown in the drastically decreased landing of Malaysian mahseer from the inland capture [12,13].

The mahseer is regarded as the most expensive freshwater food fish in Malaysia as it has high market demand and value. The price can go as high as USD 80–200 per kg though the price of cultured mahseer is not as high as that of wild-caught mahseer, which can fetch about USD 250 per kg [14]. The retail price for cultured mahseer ranges between USD 30–50 per kg [15]. Mahseer price also varies based on its source and use of the fish as food or ornamental fish.

With successful hatchery production and its ready acceptance of artificial feed, the Malaysian mahseer is now considered a good prospect for aquaculture. Lately, it has gained much attention and popularity among local farmers in Malaysia [16,17]. *Tor* spp. is cultured mainly in ponds in Peninsular Malaysia and cement tanks and cages in East Malaysia. The states of Kelantan and Sarawak are the lead producers of Malaysian mahseer. The annual production of Malaysian mahseer has been approximately 20 tonnes per year since 2012 [12,13,15,18,19,20,21,22]. This large-sized riverine fish can grow up to 30–50 kg. However, the growth rate of this fish in culture conditions is relatively slow compared to other aquaculture species [23]. It usually takes up to three years to reach the marketable size of 1.5–2.0 kg. The commercial culture of mahseer fish has also gained popularity in Indonesia, especially in the Aceh province [24,25].

The availability of polymerase chain reaction (PCR)-based microsatellites at a reasonable cost offers an easy and convenient way to generate rapid, reproducible and high throughput results for genetic diversity assessment. Microsatellites are also known as simple sequence repeat (SSR). SSR loci are hypervariable genetic markers. This feature made SSR the ideal choice for estimating genetic relatedness without prior knowledge of pedigree information [26,27,28,29,30]. Microsatellite variations are independent of natural selection because most of the SSR markers are from the non-coding regions of the genome. Therefore, they are ideal genetic markers for conservation genetics and sustainable fisheries management purposes [31]. SSR markers have been used to determine the population structures of various mahseer species in the region [32,33,34,35,36,37,38]. Population genetic structure of *T. tambroides* from several natural populations in Malaysia had been examined using SSR markers in one of the studies by [33]. Still, the study locations were slightly different from the present study. Furthermore, most of the samples used in that study were collected during the 2000s.

Broodstock development is the most vital element in developing a species for aquaculture. In usual practice, broodfish are collected from various sources for broodstock development. A detailed genetic background, such as the status of the genetic diversity and the population structure, including the demographic aspects (bottleneck and effective population size and number of migrants) and population assignment of the candidate broodstock, is often unknown. Very often, genetic studies will only be pursued for the collection after the collected broodstocks are established. Sperm cryo-banking was carried out for this indigenous *Tor* spp. as part of the conservation measure. In our research, Malaysian mahseer comprising a mixture of *T. tambra* and *T. tambroides* (and thus is addressed as *Tor* spp. in the study) were collected from 11 localities in Malaysia for broodstock development and sperm cryo-banking. Information on the genetic status of these *Tor* stocks is essential to formulate appropriate strategies for genetic resource protection and utilization in aquaculture development. However, no genetic assessment had been done on these *Tor* spp. stocks. Therefore, the genetic background, whether these *Tor* spp. stocks from different geographical regions possess different genetic makeups or whether any changes in their genetic variation over time, was unknown. It would be a waste of cost and space to maintain too many stocks of low genetic variabilities in the conservation and breeding programmes. Therefore, the main objectives of this study were to assess the genetic diversities, population structures, genetic relatedness, and their demographic aspects; and establish the genetic profiles of the *Tor* spp. collection by employing SSR markers.

## 2. Materials and Methods

### 2.1. Collection of Study Materials

**Figure 1:**
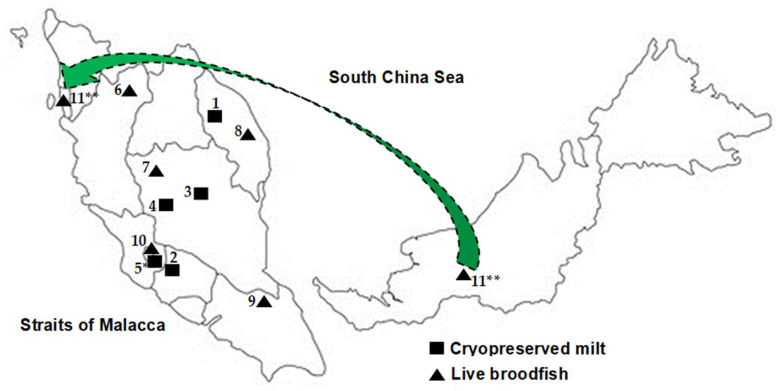
The map of the study locations where the *Tor* spp. samples were collected in this study. 1. Fisheries Research Institute Glami Lemi stock (FFRC); 2. Kg Esok, Jelebu, Negeri Sembilan (KENS); 3. Aquaculture Extension Center, Perlok, Jerantut, Pahang (PPAP); 4. Agro Harvest, Raub Ptd. Ltd., Pahang (AGHR); 5. Kelah World Ptd. Ltd., Hulu Langat, Selangor (HLKW); 6. Grik, Perak (GPRK); 7. Raub, Pahang (PHG); 8. Terengganu (TGN); 9. Mersing, Johor (MSJ); 10. Hulu Langat, Selangor (HLS); 11. Empurau, Sarawak (EMS). * This is a different *Tor* species from the stocks in Malaysia and morphologically most likely of the *T. tambroides*. The stocks from other localities were *T. tambra*; ** Fish sample was obtained from a local fish trader at Penang who claimed the stock was originated from Sarawak.

A total of 181 *Tor* spp. samples comprised 91 cryopreserved milt samples (collected from five localities for cryo-storage since 2008), and 90 scale samples of live broodfish (collected between 2010–2017 from six localities) were obtained for this study. These study samples were maintained at the Fisheries Research Institute Glami Lemi (FRIGL), Jelebu, Negeri Sembilan, Malaysia. Each broodfish was tagged with a passive integrated transponder tag. The male broodfish from which their milt samples were collected for cryopreservation in 2008 were obtained from the wild stocks of five localities caught in 2000–2008. The cryopreserved milt samples were obtained from stocks of FRIGL (FFRC, 11 samples), Kg Esok, Jelebu, Negeri Sembilan (KENS, 41 samples), Aquaculture Extension Center, Perlok, Jerantut, Pahang (PPAP, 13 samples), AgroHarvest, Raub, Pahang (AGHR, 5 samples) and Kelah World, Hulu Langat, Selangor (HLKW, 21 samples). FRIGL stock was collected from Kenyir Lake, Terengganu during FFRC, Batu Berendam, Melaka. KENS samples were collected from Kenaboi River, PPAP samples were collected from Pahang River, AGHR samples were collected from Keniam River, Taman Negara. In contrast, HLKW samples were obtained from a farmer who ran a farm at Hulu Langat and merely imported mahseer from Sumatra, Indonesia. The stocks from all ten localities in Malaysia were *T. tambra*, while samples of HLKW was *T. tambroides*. As claimed by the farm owner, samples of HLKW were *T. tambroides*. Morphologically HLKW stocks were slightly different from other stocks collected in Malaysia and most likely of *T. tambroides* [8,10]. Live broodstocks of Grik, Perak (GPRK, 16 samples), Terengganu (TGN, 14 samples) and Raub, Pahang (PHG, 11 samples) populations were collected from the wild stocks at fingerlings stage and domesticated in the pond until reaching a matured size. GPRK samples were collected from Kejar Banding River, Perak, TGN samples were collected from Berang River, Terengganu, and PHG samples were collected from Jerai River, Pahang. For Hulu Langat, Selangor (HLS, 14 samples), Mersing, Johor (MSJ, 28 samples), and Sarawak (EMS, 1 sample) populations, stocks were obtained from the local fish traders of respective localities. For EMS, five fish were collected from a local fish trader at Penang who claimed the stock was originated from Sarawak. However, only one fish survived after the quarantine period, and the sample was used in the present study. There was no overlapping in the sources of the fish among the frozen milt and scale samples. These samples were used for total genomic DNA extraction, and, subsequently, the extracted DNA samples were used for SSR genotyping. The number of samples and their sources of origin from each population are summarized in Table 1 and illustrated in Figure 1.

### 2.2. Total Genomic DNA Extraction

Each extraction reaction used 400 µL thawed milt and 0.04 g scale sample. Cryopreserved milt samples in 0.5 mL IMV straws (IMV Technologies, L’Aigle, France) were thawed at 40 °C for 7 s. After that, both ends of the IMV straws were cut, and the thawed milt sample was drawn into a 1.5 mL centrifuge tube. The frozen-thawed milt sample was precipitated by centrifugation at 10,000 rpm for 15 min. The supernatant was discarded, and the sperm pellet was then subjected to total genomic DNA extraction by using the DNeasy^®^ Blood and Tissue Kit (QIAGEN Minden, Hilden, Germany) according to the manufacturer’s instruction.

The extracted total genomic DNA samples were subjected to electrophoresis on a 1% agarose gel. Electrophoresis was carried out in 1× TAE buffer (40 mM Tris, 20 mM acetic acid, 1 mM EDTA, pH 8.0) at 80 V for 30 min to determine DNA integrity. The concentration of the extracted DNA was quantified by a NanoDrop^®^ ND-1000 Microvolume Spectrophotometer (Thermo Fisher Scientific, Inc., Waltham, MA, USA). Total genomic DNA samples were homogenized at 10 ng/µL and stored at −20 °C until use.

### 2.3. PCR Amplification

DNA was amplified by PCR. A total of 24 microsatellite primer pairs (Supplementary Materials, Table S1) were tested in this study, and suitable primer pairs, i.e., with successful amplification and produced fragments at the targeted sizes, were selected and used in the genotyping analysis. Fourteen pairs (NY01-NY14) were specifically for *T. tambroides,* according to [38], and ten primer pairs (BS01–BS10) were designed from the microsatellite DNA sequences of *T. tambroides* deposited in the NCBI genebank. Primer pairs were designed using Primer 3 version 0.4.0 software [39,40]. The 5′ end of either the forward or the reverse primer in each primer pair was labelled with a fluorescent dye (6-FAM, HEX, TAMRA, or ROX). Positive and negative controls were included in each amplification reaction. A sample with known fragment size was used as a positive control, while a sample without DNA template but substituted with nuclease-free water was used as a negative control.

The PCR reactions were performed in a total volume of 25 µL reaction mixture containing 2.5 µL 5X Go Taq^®^ Flexi Buffer (with 1.5 mM MgCl_2_), 200 µM each dNTP, 10 pmol each of forward and reverse primers, 0.5 U Taq DNA Polymerase (Promega Corporation, Madison, WI, USA) and 3–10 ng template DNA. The PCR amplification profile was started with an initial denaturation at 94 °C for 4 min, 35 cycles of 94 °C for 35 s, annealing temperature between 48–66 °C (depending on the primer pairs) for 35 s and extension at 72 °C for 1 min, followed by a final extension at 72 °C for 10 min and finally held at +4 °C. PCR was performed using MyCycler^TM^ Personal Thermal Cycler (Bio-Rad Laboratories, Inc., Hercules, CA, USA). Before proceeding with the amplification of all samples, the optimal annealing temperature of each primer pair was determined via gradient PCR reaction with the range of annealing temperatures tested from 45 °C to 70 °C.

### 2.4. Gel Electrophoresis and Fragment Analysis

The amplification specificity of the PCR products was determined before performing the fragment analysis via conventional gel electrophoresis on 1.8% agarose gel containing Invitrogen 1× SYBR^TM^ Safe DNA Gel Stain (Thermo Fisher Scientific, Inc., Waltham, MA, USA). A volume of 5 µL of the PCR products was used for the gel electrophoresis. Agarose gel electrophoresis was carried out in 1× TAE buffer at 100 V for 30 min. The gel image was then visualized and photographed under UV illumination using the G: Box Gel Documentation System (Syngene Inc., Frederick, MD, USA).

Fragment analysis was conducted via capillary electrophoresis on the PCR products generated from primer pairs labelled with different fluorescent dye colours (Supplementary Materials, Table S1). Three to four singleplex PCR products were pooled for each run. The optimum dilution factor of the PCR products was determined before the electrophoretic fragment analysis. The samples were then diluted according to the optimum dilution factor (100×–3000×), as shown in Table S2 (Supplementary Materials), and mixed with GeneScan^TM^LIZ-500^®^ size standard and Hi-Di^TM^ Formamide (Thermo Fisher Scientific, Inc., Waltham, MA, USA). After that, 2 µL of the sample mixture was loaded into the Applied Biosystems ABI3730XL Genetic Analyser (Thermo Fisher Scientific, Inc., Waltham, MA, USA) to determine the peaks. Lastly, the raw data were analyzed using the GeneMapper^®^ Software Version 4 to determine the fragment sizes and the height of the peaks.

### 2.5. Data Analysis

#### 2.5.1. SSR Genetic Diversity and Polymorphism

Microsatellite loci were scored and analyzed using the Power Marker Software Version 3.25 to determine the genetic diversity and the genetic distance of the *Tor* spp. samples in this study [41]. Genetic diversity and polymorphism were analyzed by locus, population and different sample types. The genetic statistics determined using Power Marker Software Version 3.25 were major allele frequency (MAF), number of alleles per locus (N_A_), number of genotypes (N_G_), expected heterozygosity (He), heterozygosity (Ho), inbreeding coefficient (*f*) and the polymorphism information content (PIC) [41]. The GenAleX 6.51b2 software was used to determine allelic richness (A_r_), effective number of alleles (A_e_), number of private alleles (A_p_) and percentage of polymorphic loci [42]. Markov chain exact tests for conformance to Hardy Weinberg Equilibrium (HWE) were carried out on the *p*-values following [43], with sequential Bonferroni correction, according to [44]. Genotyping errors due to null alleles, stuttering and large allele dropouts were analysed using the MICROCHECKER version 2.2.1 software for all loci and populations except EMS [45]. The SSR data of the EMS population, with only one sample, were insufficient for the analysis.

#### 2.5.2. Population Differentiation, Genetic Distance and Genetic Structure

The genetic structure of the *Tor* spp. collection was assessed via analysis of molecular variance (AMOVA), F-statistics, pairwise population comparisons and population differentiation, determined using the ARLEQUIN software version 3.5.2.2 [46]. AMOVA was performed based on the distance method with 10,000 permutations [47]. F-statistics analysis was performed based on standard permutations across the full data set of allelic distances to assess the genetic differentiation of all the loci in all populations [48]. Population comparisons were evaluated by computing pairwise population differentiation estimates (F_ST_) between populations based on the distance method, with the significance level set at *p* = 0.05 [49]. Population differentiation was determined via an exact test with 100,000 steps in a Markov chain and 10,000 dememorization steps based on genotype frequencies. The overall differentiation of the *Tor* spp. collection was determined using a variant of the Mantel test, with the genetic distance matrix constructed based on the shared allele distance for each pair of individuals. Principal coordinates analysis (PCoA) for the distribution of each individual in all populations and estimation of gene flow (Nm) was performed using GenAleX 6.51b2 software [42]. PCoA was made using the first and second components based on the co-dominant genotypic distance matrix. Gene flow among populations was estimated using an indirect method based on the number of migrants per generation (Nm) [50].

Both distance-based and Bayesian model-based clustering methods were used to determine the population structures of the *Tor* samples studied. The distance-based method through Nei’s genetic distance implemented in Power Marker Software Version 3.25 was performed to construct an Unweighted Pair Group Method with Arithmetic Mean (UPGMA) dendrogram and viewed using the TreeView v 1.6.6 program [41,51,52]. The Bayesian model-based clustering method was analyzed using STRUCTURE Version 2.3.4 [53]. In the Bayesian model-based approach, the true number of clusters (K) in the samples studied was determined by computing the log-likelihood Ln P(D) with K values varying from one to eleven and with 20 independent runs for each K value. The most appropriate K value was determined with a 25,000 burn in period and 25,000 Markov Chain Monte Carlo (MCMC) replications after burn in, using the admixture model with no prior population information. The optimal number of clusters is the one that corresponds to the highest value of delta K (ΔK). ΔK is based on the rate of change in the log probability of data between successive K values following the criteria of [54]. The output from STRUCTURE software was then visualized and parsed using the web-based Structure Harvester program [55].

#### 2.5.3. Genetic Relatedness

Genetic relatedness between and among individuals within each population was assessed by seven relatedness estimators implemented in the COANCESTRY Version 1.0.1.9 software based on an individual’s multilocus genotype data [56]. The relatedness estimators used were five-moment estimators denoted as Wang [57], LynchLi [58,59], LynchRd [60], Ritland [61], QuellerGt [62], and two likelihood estimators denoted as TrioML [63] and DyadML [64]. The moment estimation is based on calculating the level of shared alleles between sample pairs, while the likelihood estimation classifies samples to a limited number of related classes. Inbreeding is assumed to be absent in the all-moment estimators used. The genotyping error rate was zero for all loci. A bootstrap analysis (1000 bootstrap replicates) was conducted to test the statistical differences between populations in mean relatedness coefficients (Rxy). The difference between populations is significant at a 95% confidence level when the difference in mean relatedness between the two groups lies outside the 2.5 and 97.5 percentiles of the distribution curve obtained by bootstrapping [56].

#### 2.5.4. Population Bottleneck, Effective Population Size (Ne), and Population Assignment

Evidence of a recent bottleneck for each population except EMS was tested for using BOTTLENECK version 1.2.02 [65]. Three mutation models, i.e., the infinite alleles model (IAM), two-phase model (TPM), and stepwise mutation model (SMM), were used in the analysis for heterozygosity excess. The TPM model was applied with a variance of 30 and a stepwise mutation probability of 70%. The results over loci for the significant presence of an excess of observed heterozygotes were derived from the Wilcoxon sign-rank test [66]. The allele mode shift test, i.e., distortion from L-shape allele distribution within each population indicated a recent bottleneck occurrence, was also tested for using the same software with 1000 iterations.

The effective population size (Ne) was estimated based on linkage disequilibrium data at unlinked loci according to the Burrows method with a bias correction using the *LDNE* Software version 1.31 [67,68]. The critical value (P_crit_) was defined at 0.01, i.e., all alleles with frequencies less than 0.01 were excluded from the analysis. The 95% confidence intervals for Ne were determined by the JackKnife method. Population assignment was also performed using GenAleX 6.51b2 software [42] to detect levels of genetic admixture according to allele frequencies following [69,70].

## 3. Results

### 3.1. SSR Genetic Diversity and Polymorphism

#### 3.1.1. Genetic Diversity by Population

Among the eleven populations studied, Kelah World, Hulu Langat, Selangor (HLKW) produced the highest N_A_ (146), A_r_ (6.6818), allele numbers per locus (1–20), and the number of genotypes (7.8182), while EMS generated the lowest N_A_ (28), A_r_ (1.2727), allele numbers per locus (1–2) and the number of genotypes (1.0000) (Table 2). The mean values of A_r_ and the number of genotypes were 3.8967 and 4.8682, respectively. The MAF ranged from 0.5097 (HLKW) to 0.8636 (EMS) and with a mean value of 0.6495. The number of effective alleles (A_e_) generated varied from 1.273 to 3.792, with a mean of 2.543. The highest Ae was seen in the AgroHarvest, Raub, Pahang (AGHR) population, while the lowest A_e_ was in the EMS population. A total of 87 private alleles unique to a single population were produced from the *Tor* samples, with the highest number from the HLKW population (52 alleles), followed by the Aquaculture Extension Center, Perlok, Jerantut, Pahang (PPAP) population (11 alleles). Private alleles were generated in nine populations, i.e., 81.8% of the populations, while they were absent in the AGHR and HLS populations. The list of private alleles generated in each population is shown in Appendix A.

The percentages of polymorphic loci ranged from 27.27% in the EMS population to 95.45% in PPAP, HLKW and TGN populations, with an average of 76.45%. The lowest genetic variation was in the EMS population due to its small sample size. The highest He (0.5970), Ho (0.4545) and PIC (0.5711), respectively, were shown in HLKW, while the lowest He (0.0682), Ho (0.2727) and PIC (0.1023), respectively, were seen in EMS. The mean values of He, Ho and PIC, were 0.4189, 0.4083 and 0.3966, respectively. In this study, six out of the eleven populations with He < Ho were conforming to HWE.

#### 3.1.2. Level of Inbreeding across Loci and Populations

The inbreeding coefficient across loci (*f*) ranged from −0.6226 (BS08) to 0.9238 (NY13) and with an average *f* at 0.2259 (Table 3). Positive *f* was showed in sixteen loci, while negative *f* was detected in six loci. Positive *f* values indicated excess homozygosity and the occurrence of deviations from HWE in these loci. On the contrary, negative *f* values indicated an excess of observed heterozygotes and no incident of inbreeding. The inbreeding coefficient across populations (F_IS_) was in the range of −0.003 to 0.234, with a mean of 0.015 (Table 2). The inbreeding coefficient was the highest in the HLKW population (0.234). Positive F_IS_ was detected in six populations, while negative F_IS_ was detected in five populations. The mean F_IS_ of 0.015 within the populations studied was small, indicating a low level of inbreeding.

#### 3.1.3. Null Allele

As revealed by the analysis using the MICROCHECKER software, a general excess of homozygotes (heterozygote deficits) for most allele size classes might be present in sixteen loci (72.7%), suggesting the presence of null alleles. For those loci with null alleles, the total observed homozygotes appeared higher than the total expected homozygotes and exhibited positive *f* values (Table 3). Null alleles were detected in nine out of the eleven populations studied (Supplementary Materials, Table S3). The null alleles detected in each population for the respective SSR markers are summarized in Appendix (Table A1). The AGHR population showed no evidence for null alleles in all loci as the total expected homozygotes appeared higher than the total observed homozygotes.

Meanwhile, stuttering, which is indicated by a highly significant shortage of heterozygous genotypes with alleles of one repeat unit difference, might have occurred in thirteen loci (59.1%) in this study. The HLKW population showed the highest occurrence of null alleles and stuttering. Nevertheless, the results showed no evidence for large allele dropout in all loci. Large allele dropout occurs when large alleles do not amplify as efficiently as small alleles.

### 3.2. Population Differentiation, Genetic Distance and Genetic Structure

#### 3.2.1. Genetic Differentiation

The level of genetic differentiation, as revealed by AMOVA, indicated that the variation among populations (V_a_) was 14.92%, variation among individuals within populations (V_b_) was 9.00%, and variation within individuals was 76.09%. These indicated that the *Tor* spp. collection was highly variable, and, statistically, all these variations were highly significant (*p* < 0.001) (Table 4).

The estimates of fixation indices from the F-statistics analysis were with mean values of overall F_ST_, F_IS,_ and F_IT_ of 0.149, 0.106, and 0.239, respectively. All the three indices exhibited statistically highly significant differences (*p* < 0.001) from zero among and within all populations indicating the phenomenon of inbreeding. Inbreeding was resulting in a loss of heterozygosity among populations (F_ST_), among individuals relative to the subpopulation (F_IS_), as well as within individuals relative to the total population (F_IT_).

Pairwise F_ST_ values ranged from 0.016 to 0.237 among populations, indicating mixed levels (from low to high) of genetic differentiation among the populations (Supplementary Materials, Table S4). The lowest pairwise F_ST_ value (0.016) was between the MSJ and HLS populations. The highest value (0.237) was between the GPRK and KENS populations. Significantly higher (*p* < 0.05) genetic differentiation than all other populations was observed in three populations, i.e., HLKW, GPRK, and KENS. The pairwise F_ST_ values ranged from 0.135 to 0.235 between the HLKW population and other populations, ranged from 0.092 to 0.237 between the GPRK population and other populations, and ranged from 0.090 to 0.237 between the KENS population and other populations. For EMS (*T. tambra* from Sarawak), significant population differentiation was observed between EMS and HLKW, GPRK and KENS populations. Still, it was not significant between the EMS population and seven other populations (i.e., AGHR, FFRC, HLS, MSJ, PHG, PPAP and TGN). Besides the HLKW population, high population differentiation, with pairwise F_ST_ greater than 0.2, was also observed between the KENS and EMS populations (pairwise F_ST_ 0.235), between the EMS and PHG populations (pairwise F_ST_ 0.227), between the AGHR and PHG populations (pairwise F_ST_ 0.211), and between the GPRK and PHG populations (pairwise F_ST_ 0.210). Pairwise populations with F_ST_ < 0.05 indicated that the genetic differentiation was small and insignificant between them. For them, the allele frequencies within each population pair were very similar. These were seen in the pairwise populations of HLS-MSJ (F_ST_ 0.016), FFRC-TGN (F_ST_ 0.050), and AGHR-TGN (F_ST_ 0.036).

#### 3.2.2. Genetic Distance

Genetic divergence among the populations, as revealed by pairwise Nei’s (1983) genetic distances, ranged from 0.062 to 0.423 among the populations (Supplementary Materials, Table S4). The lowest genetic distance (0.062) was between the MSJ and HLS populations, while the highest genetic distance (0.423) was between the HLKW and EMS populations. Generally, HLKW and EMS showed genetic divergence ranged from 31.8–42.3% and 27.5–42.3%, respectively, compared to other populations. Other pairwise populations, i.e., TGN and AGHR, TGN and FFRC, and KENS and PPAP, revealed genetic divergence < 15%. Small genetic distances between these populations indicated that the populations were genetically closely related.

#### 3.2.3. Genetic Structure

**Figure 2:**
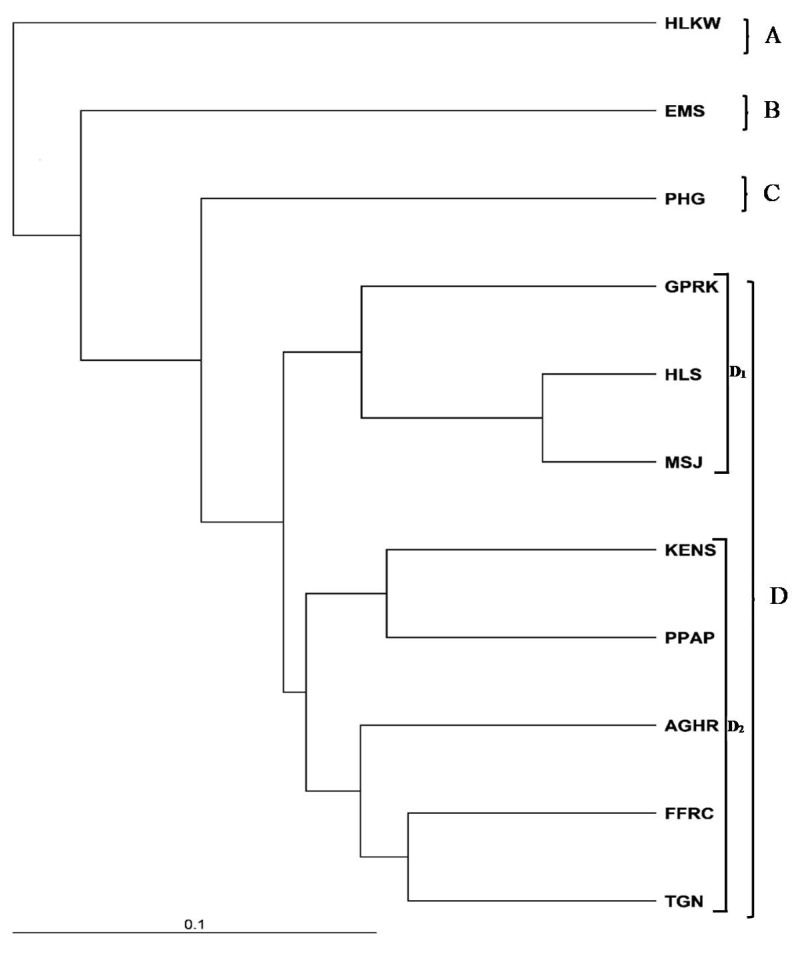
UPGMA dendrogram generated based on Nei’s (1983) genetic distance; HLKW: Kelah World, Hulu Langat, Selangor; EMS: Empurau, Sarawak; PHG: Raub, Pahang; GPRK: Grik, Perak; HLS: Hulu Langat, Selangor; MSJ: Mersing, Johor; KENS: Kg Esok, Jelebu, Negeri Sembilan; PPAP: Aquaculture Extension Center, Perlok, Jerantut, Pahang; AGHR: AgroHarvest, Raub, Pahang; FFRC: FRIGL stock (collected during FFRC Batu Berendam, Melaka); TGN: Terengganu.

Based on the UPGMA dendrogram generated based on the Nei’s genetic distance (1983), as shown in Figure 2, the samples were divided into four main clusters, i.e., (A) HLKW, (B) EMS, (C) PHG, and (D) a large cluster comprising of eight populations. Cluster D was further sub-divided into two sub-groups. The first sub-group (D_1_) consisted of three populations (GPRK, HLS and MSJ). The second sub-group (D_2_) comprises five populations (KENS, PPAP, AGHR, FFRC and TGN), of which four populations were the localities from which the cryopreserved milt samples were obtained. Based on the population structure dendrogram generated, it was evident that the populations within subgroups D_1_ and D_2_ were closely related, as revealed by the small Nei’s genetic distances (1983) (Supplementary Materials, Table S4).

The principal coordinates analysis (PCoA) plot was also generated to show the genetic structure among all the *Tor* spp. samples. The results showed that the first, second and third component axes accounted for 28.50%, 11.88% and 6.99%, respectively, of the total variance, and the eigenvalues were 190.80, 79.50 and 46.79, respectively. The PCoA plot showed that the *Tor* spp. samples were in four distinct groupings, with the first principal component differentiating the HLKW population from the rest of the samples on the left quadrant. In contrast, the remaining populations formed three distinct groups on the right quadrant (Supplementary Materials, Figure S3). Samples with identical SSR genotypes were superimposed on each other in the plot. Generally, locality-specific clusterings were evident on this PCoA plot, although some of the samples were not grouped tightly in the HLKW, MSJ and HLS populations. The samples of these three populations were split into two distinct fragmentary clusters instead. Overall, there were four genetically different groups formed among the *Tor* spp. samples studied. These groups were: (I) EMS and GPRK; (II) PHG and KENS; (III) AGHR, TGN, FFRC, PPAP, MSJ and HLS; (IV) HLKW.

**Figure 3:**
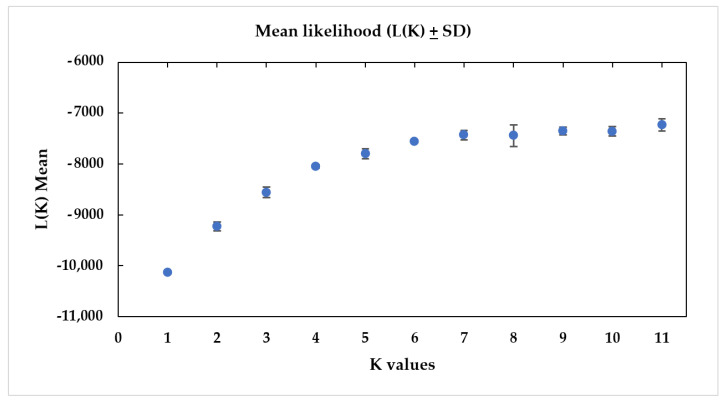
Plot of mean likelihood L(K) and variance per K value from STRUCTURE on 181 *Tor* spp. samples genotyped for 22 polymorphic microsatellite loci. Mean L(K) was obtained over 20 independent runs for 11 different K values.

The model-based cluster analysis based on the magnitude of delta K (ΔK) statistics generated the highest K value at K = 4 (Supplementary Materials, Figure S4). Therefore, the most likely number of clusters to best explain the *Tor* spp. population structure was four. Another peak was also observed at K = 6. From the plot of mean likelihood L(K) and variance per K value generated, the likelihood L(K) values over 20 runs were consistent without showing significant variance for the same K value (Figure 3). 

**Figure 4:**
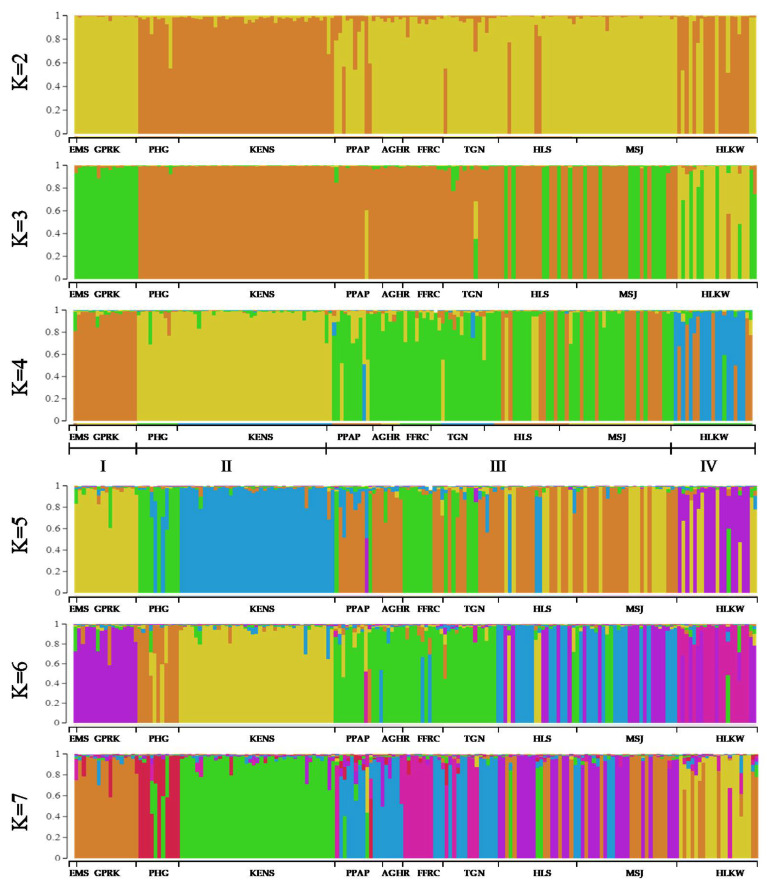
Population structure of the *Tor* spp. individuals collected from eleven populations estimated according to the Bayesian model by the STRUCTURE program Version 2.3.4 for K = 2–7 based on 22 microsatellite loci. Different colours indicate different genetic clusters: EMS: Empurau, Sarawak; GPRK: Grik, Perak; PHG: Raub, Pahang; KENS: Kg Esok, Jelebu, Negeri Sembilan; PPAP: Aquaculture Extension Center, Perlok, Jerantut, Pahang; AGHR: AgroHarvest, Raub, Pahang; FFRC: FRIGL stock (collected during FFRC Batu Berendam, Melaka); TGN: Terengganu; HLS: Hulu Langat, Selangor; MSJ: Mersing, Johor; HLKW: Kelah World, Hulu Langat, Selangor.

Comparisons of the proportional membership of the *Tor* spp. individuals estimated by the STRUCTURE software at varying K values from K = 2 to K = 7 are shown in Figure 4. Generally, low gene flow was observed among the populations (individual q values > 0.8), except in the HLKW and PPAP populations, which showed admixed ancestry for some of the samples.

At K = 4, the eleven populations of *Tor* spp. were unambiguously divided into four groups with unique and distinct genetic compositions corresponding to the respective geographical regions. The four different groups were: (I) EMS, GPRK (Northern); (II) PHG, KENS (East Coast, Pahang River); (III) PPAP, AGHR, FFRC, TGN, HLS, MSJ (East Coast, Terengganu River); and (IV) HLKW (Indonesia). All members in EMS, GPRK (group I), PHG, KENS (group II), and AGHR, FFRC, and TGN (group III) were correctly assigned to their respective populations. Nevertheless, some of the individuals in both the HLS and MSJ populations of group III were assigned to group I (30% members of HLS and 35.7% members of MJS, respectively). Individuals in the PPAP and TGN populations, 76.9% and 92.9%, respectively, were assigned to group III. Some members in the PPAP population had admixed ancestry with group II and group IV, while 7.1% of the members in the TGN population had admixed ancestry with group IV. Meanwhile, some members in the HLKW population were found assigned to group I and showed admixed ancestry (individual *q* values < 0.8) with the *Tor* spp. from Northern Peninsular Malaysia.

At K = 6, it was observed that population differentiation occurred in groups II and III. PHG and KENS populations of group II were differentiated into two distinct groups. HLS and MSJ populations were differentiated from group III to form another separate group.

### 3.3. Genetic Relatedness among Individuals

The genetic relatedness among individuals in all populations was successfully generated from seven relatedness estimators implemented in the COANCESTRY program. Table 5 shows the mean genetic relatedness among individuals of each population generated from each estimator. Generally, the relationships among samples were consistent across all estimators. The likelihood estimators revealed higher estimates than the moment estimators. For the moment estimators, positive genetic relatedness (Rxy) was only observed in the KENS and PHG populations using the [66,67,68] estimators. The highest relatedness values with mean Rxy 0.053 (LinchLi) were observed in the KENS population, indicating that the samples in this population were genetically more related than in the other populations. In most populations, the values of mean relatedness based on moment estimators appeared as negative values. A negative relatedness estimate means the individuals are less related than the average relatedness. The bootstrap analysis also revealed no statistical differences between populations in mean relatedness coefficients (Rxy) for all estimators (Table 5).

The correlation coefficients between each pair of the seven relatedness estimators were calculated to compare different estimators in all the *Tor* populations. Overall, the relatedness estimators were positively correlated with correlation coefficients ranging from 0.531 to 0.986 among all populations. The range of correlation coefficients in each population is summarized in Table 5.

### 3.4. Analysis for Bottleneck, Effective Population Size (Ne) and Population Assignment

#### 3.4.1. Bottleneck Analysis

The results from bottleneck analysis using IAM suggested that there were occurrences of bottlenecks in six populations, i.e., FRIGL stock (FFRC), GPRK, HLKW, Kg Esok, Jelebu, Negeri Sembilan (KENS), MSJ and PHG (Table 6, Supplementary Materials, Table S3). Analysis using TPM and SMM showed evidence of a recent reduction in population size within the population PHG, which supported the bottleneck phenomenon in this population. Nevertheless, there was no evidence of a recent bottleneck within three populations, namely HLS, PPAP and TGN. A mode-shift in the allele frequencies was detected in the AGHR population under mutation-drift equilibrium. However, there were no significant bottlenecks in all the mutational models in this population (Supplementary Materials, Table S3). The allele frequency distributions were approximately L-shaped in all the other populations, indicating no mode-shifts in the allele frequencies.

#### 3.4.2. Estimation of Effective Population Size (Ne)

The estimates of effective population size (Ne) within each population determined from the linkage disequilibrium data are listed in Table 7. The Ne values were negative for the AGHR, EMS and GPRK populations. Negative estimates of Ne indicate genetic drift. However, there was no evidence for any disequilibrium caused by genetic drift due to the finite numbers of parents in these populations. Therefore, sampling errors and small sample sizes in particular populations such as EMS and AGHR might have contributed to this phenomenon (Supplementary Materials, Table S3).

#### 3.4.3. Population Assignment

The outcome of the population assignment showed that 90% (163) of the individuals from the eleven populations were assigned correctly to the self-population, i.e., their original sampling locality. In comparison, only 10% (18) individuals of four populations were mismatched and assigned to other populations (Table 7). Out of the four mismatches in HLKW, two individuals each were assigned to MSJ and GPRK. Out of the four mismatches in HLS, two individuals each were assigned to KENS and GPRK. Among the seven mismatches in MSJ, three individuals each were assigned to HLS and TGN, while one individual was assigned to AGHR. For the three mismatches in TGN, two individuals were assigned to FFRC, while one individual was assigned to AGHR. The seven populations with 100% correctly assigned individuals were AGHR, EMS, FFRC, HLS, KENS, PHG, and PPAP.

## 4. Discussion

In this study, the total genomic DNA extracted from both the frozen milt and scale samples of *Tor* spp. produced identical SSR loci with the expected sizes (Supplementary Materials, Figures S1 and S2). Similar findings were also obtained in the study on Whitefish (*Coregonus lavaretus* L.) using DNA from adipose fins and cryopreserved milt [71]. SSR alleles were also proven to be the same from different tissues of the same individual, as reported in a study of Chinese Holstein bulls using blood and semen samples [72]. Therefore, the total genomic DNA obtained from different sample types should not be disputed for its abilities to amplify similar and consistent PCR products.

### 4.1. Genetic Diversity of the Tor spp. Collection

The *Tor* collection in this study still retained a reasonable amount of genetic variation, with allele richness of 1 to 33 alleles per locus, polymorphic loci 76.45%, and an average PIC of 0.4942. Compared to studies on the same species by [32,34,36], the microsatellite loci assessed in the present study showed the highest polymorphism level and allelic richness. The number of alleles was influenced by the sample sizes [73,74]. Generally, the larger the sample size, the higher the number of alleles generated from the population. Allelic richness, corrected for unequal sample size among samples for each locus, is the preferred measure for genetic diversity.

Allelic richness is an essential element in conservation programmes, as it is indicative of a population’s long-term potential for adaptability and persistence [75,76]. The allelic richness of the HLKW population (1–20 alleles per locus) obtained from Indonesia, believed to be *T. tambroides*, was quite similar to the allelic richness, i.e., 5–21 alleles per locus obtained on the same species in the study by [36]. For *T. tambra* in all the other populations that we studied, the allelic richness ranged from 1–2 alleles per locus to 1–17 alleles per locus, but with most populations (i.e., FFRC, PPAP, AGHR, PHG, EMS and GPRK) having the allelic richness of around ten alleles per locus. The reported allelic richness for *T. tambra* was ten alleles per locus in several studies [32,34,36].

Most SSR loci (90.9%) revealed a significant deviation from HWE after the Bonferroni correction. Departures from HWE in most SSR loci are plausible and have been commonly reported in studies of natural populations in a wide range of fish species [77,78,79,80,81]. Excess or lack of heterozygotes can cause departures from HWE. In this study, heterozygote deficiencies were observed in sixteen loci, whereas an excess of heterozygotes was detected in six loci. Heterozygote deficiency can be attributed to small sample size, the presence of inbreeding or genetic patchiness (Wahlund effect), reduction in effective breeding population size (Ne), and the presence of null alleles, which caused an excess of homozygotes. Small sample size may cause the founder effects, bottleneck effects or genetic drift [35,82]. Selective breeding, over-exploitation and anthropogenic disturbances resulted in occurrences of inbreeding and reduction in Ne [83,84,85]. The high mutation rate in microsatellites increases the occurrence of null alleles [86]. In this study, the small sample sizes of the AGHR and EMS populations (with <10 samples) could result in sampling error, inbreeding and the presence of bottleneck, all of which could cause heterozygote deficits, resulting in deviations from HWE and subsequently resulting in population differentiation.

The small sample size in both EMS and AGHR populations has resulted in low genetic variation. In populations with small sample sizes, rare genotypes are likely to be included in the samples. The small population size also increases inbreeding and genetic drift, thus reducing genetic variability over the long term [87]. When inbreeding occurs, the number of homozygotes will increase because the mating individuals have the same alleles. This excess homozygosity, in turn, causes heterozygosity deficit in the population [88,89,90,91].

Heterozygosity deficit in the present study could also be the consequence of null alleles or stuttering among the SSR loci. Both null alleles and stuttering were detected in nine populations in this study. Of the nine populations with null alleles, five populations, i.e., HLKW, MSJ, HLS, FFRC and PPAP, showed heterozygote deficits. A highly significant (*p* < 0.001) heterozygosity loss among and within the populations was also revealed in the present study, as revealed in the AMOVA and F-statistics analysis. For populations with null alleles in the present study, the null alleles’ frequencies were relatively high in general (9.1–30.9%, data not shown). These populations showed highly significant genetic differentiation, with low gene flow among the populations as shown from the AMOVA.

Each locus that deviated from HWE can amplify at least one allele in all the samples. Thus, the low frequencies of null alleles were not enough to affect the analysis [92]. Generally, null alleles with low frequencies between 5% and 8% would only have a minor effect on the classical estimation of population genetic parameters such as genetic diversity, population differentiation, population FST and genetic distances. However, when null alleles were present at frequencies higher than 10%, it could affect the genotyping of individuals at some loci and lead to the under-estimation of genetic diversity and the over-estimation of population differentiation. Genetic distances tend to be underestimated when null alleles occurred at high frequencies [93]. The null allele at microsatellite loci with frequencies higher than 10% and its consequences in estimating population structure and differentiation have been reported in several studies on bivalve species [94,95,96,97,98]. Nevertheless, in a study on Wedge Clam (*Donax trunculus*), the presence of unusually high frequency of null alleles (>10%) did not appear to affect the FST estimates significantly [94].

The presence of null alleles in microsatellite data and their consequences on population genetic parameters had been tested using various analytical and simulation tools [99] and actual population samples [94]. As shown in the simulations by [100], those SSR loci with null alleles would slightly overestimate the FST but are unlikely to impact genetic differentiation significantly. Therefore, SSR loci with null alleles that did not seem to alter the overall outcome of assignment testing could still be included in the studies. In this study, 163 out of 181 (90%) individuals were correctly assigned to their respective populations. Five out of nine populations with null alleles AGHR, EMS, FFRC, GPRK, KENS, PHG and PPAP exhibited 100% correctly assigned individuals. Therefore, all 22 SSR loci were kept and used in the present study.

### 4.2. Genetic Differentiation and Genetic Structure Analysis

In the present study, the fixation indices F_ST_, F_IS_ and F_IT_ indicated a significant reduction in heterozygosity within and among the populations due to non-random mating. F_IS_ values significantly higher or lower than zero reveal inbreeding or outbreeding, respectively [101]. As reflected in the AMOVA, genetic differentiation was at a medium level in the overall *Tor* spp. collection (F_ST_ = 0.149), as evidenced by the low level of gene flow estimate (N_m_ = 1.548 per generation) and highly significant level (*p* < 0.001) of inbreeding among individuals within population (F_IS_ = 0.106). Generally, it was observed in this study that the inter-population differentiation was low among the *Tor* spp. populations in Malaysia. Similar findings were also reported in the previous study by [33] on the same species. Nevertheless, a mixed level of population differentiation, from low to high, was observed among the *Tor* populations, with the pairwise population F_ST_ values ranging from low (0.016) to very high genetic differentiation (0.237) following the F_ST_ classification by [102]. Significant differences (*p* < 0.05) were detected in 83.6% of the pairwise comparisons among populations. These confirmed their population divergence.

Genetic differentiation can be attributed to migration, geographical barriers, genetic drift and gene mutation [103,104]. Low gene flow resulted in small genetic variation transfer from one population to another among the *Tor* spp. collection. The proportional membership of *Tor* spp. individuals with low genetic admixtures (individual *q* value > 0.8), as shown in Figure 4, revealed a low level of gene flow. Since the Nm value across the overall population in this study was greater than one, it was likely that genetic drift was not the main factor accounting for genetic differentiation among the *Tor* spp. populations. From the pairwise F_ST_ generated for the *Tor* spp. populations, results presented relatively higher differentiation between HLKW and all other populations, indicating that the population is most likely a separate species (*T. tambroides*). The same observation was also seen in the EMS population, which revealed significantly higher differentiation between HLKW, KENS and GPRK populations but no significant differentiation from other remaining populations. For AGHR and EMS, a small sampling size looks likely to be the main cause of population differentiation. For other populations, it seems more likely that the Wahlund effect due to geographic distances or habitat fragmentation may have caused the local genetic differentiation among the *Tor* spp. by limiting the gene flow among the populations [105,106]. Habitat fragmentation resulted in a reduction in the genetic diversity and viability of the small and isolated populations, consequently impacting the population genetic structure [107].

### 4.3. Genetic Distance and Population Structure among Sampling Locations

Genetic distance among the *Tor* spp. populations ranged from 6.2% to 42.3% in this study. A great genetic distance was observed between the HLKW population and the other populations, with a pairwise genetic coefficient ranging from 31.8% to 42.3%. This finding again supported the idea that the HLKW population was from a different *Tor* species than the other populations because there was no sexual selection between different species. The pairwise genetic coefficient of the *T. tambra* populations ranged from 6.2% to 29.3%. *T. tambra* from EMS of Sarawak also showed a higher genetic distance from other *T. tambra* populations in Peninsular Malaysia, ranging from 27.5% to 35.3%. The distinct population clusterings were further supported by the results of the population assignment tests, using both PCoA analysis and Bayesian cluster analysis. A high percentage of correctly assigned individuals indicated substantial genetic divergence among the populations [108]. The pattern of clustering using Bayesian analysis was similar to the PCoA, with four genetically distinct groups formed according to the geographical origins of the *Tor* spp. samples obtained. Moreover, the genetic admixture of all the *Tor* stocks was relatively low (individual *q* value > 0.8), indicating that individuals in each population were weakly differentiated. These genetically uncontaminated populations served as ideal sources of fresh alleles for future aquaculture and restocking programmes [27].

Similar to the genetic structures revealed in both the PCoA plot and the model-based cluster analysis at K = 4, the UPGMA clustering of *Tor* populations could also be explained according to their geographical distribution. Populations in cluster D (GPRK, HLS, MSJ, KENS, PPAP, AGHR, FFRC and TGN), as illustrated in the UPGMA dendrogram, had small genetic distances which ranged from 6.2% to 18.3%, indicating that these populations were closely related and had a recent common ancestor. It was apparent that these closely related populations were from the same source and origin. Similar clustering was also observed in the previous study by [33], i.e., samples from Negeri Sembilan, Pahang and Perak were closely related and grouped in the same cluster.

Samples of the FFRC population were obtained from Kenyir Lake, while samples of the TGN population were collected from the Terengganu River, originating in Kenyir Lake. Samples of population PPAP were collected from the Pahang River. In contrast, the samples of the KENS population were obtained from the Kenaboi River, which is one of the tributaries of the Pahang River. However, the close relationship between samples of the HLS population from the Hulu Langat River and samples of the MSJ population from Mersing Johor could not be explained according to their geographical locations because these two river systems were not from the same origin. The Hulu Langat River flowed westwards of Peninsular Malaysia and ended at the Straits of Malacca, while the Mersing River flowed to the southeast of Peninsular Malaysia and ended at the South China Sea. 

Based on the population structure derived from the STRUCTURE analysis at K = 4, it was noticed that individuals in populations HLS and MSJ had similar genetic compositions. *Tor* individuals from both the HLS and MJS populations comprise a mixture of *Tor* with distinct genetic contributions from the North (GPRK) at ≈35% and East Coast (PPAP, AGHR, FFRC and TGN) of Peninsular Malaysia at ≈65%. Generally, the samples of populations HLS and MSJ were more closely related genetically to population GPRK. This observation was also supported in the population assignment test, by which 25% and 33.3% of the HLS and MSJ populations respectively mismatched one another and mismatched with the GPRK population or populations from the East Coast (TGN and AGHR) of Peninsular Malaysia. The admixture percentages were low among individuals in the HLS and MSJ populations, indicating that they did not interbreed. Therefore, it looked more likely that the local fish traders who supplied the HLS and MSJ stocks had obtained their fish stocks from the same source.

On the other hand, the samples of AGHR, which originated from the Keniam River, were found to be more closely related to the stocks from FFRC and TGN. In the meantime, both the GPRK and EMS were found to cluster in the same grouping in both PCoA plot and Bayesian cluster analysis, indicating that samples from Perak and Sarawak were closely related. These results again highlighted the possibility of mislabeling the EMS sample by the fish trader who supplied the fish. The so-called EMS broodfish was most probably from the local source in north Peninsular Malaysia. Unfortunately, no other samples from the same population were available for verification.

### 4.4. Genetic Relatedness among Individuals

Genetic relatedness shows the relationship between individuals in a population [109]. Knowledge of the genetic relatedness of individuals in a population is important in genetic analysis to estimate heritabilities, genetic correlations and breeding values for developing optimized strategies for artificial selection and conservation [110]. The mean Rxy among all *Tor* samples revealed by different relatedness estimators in the present study were not significantly (*p* > 0.05) different among all *Tor* populations across all estimators. It was also observed that mean Rxy based on the moment estimators showed negative values in most populations. A negative Rxy value indicates that the individuals are less related than the average relatedness. It also reflected how much lower the probability of recent coalescence is for the individuals relative to the average probability for all considered individuals from the reference population [111]. Meanwhile, mean Rxy based on the likelihood estimators was slightly higher with positive values and highly correlated, especially among HLKW, HLS and MSJ populations. A similar observation was also reported in a study by [31] on seabass (*Lates calcarifer*), in which the Rxy estimates for wild and hatchery stocks did not differ significantly (*p* > 0.05). However, a significant increase of genetic relatedness with a high correlation coefficient and a decline in Ne estimates were detected within a selectively bred population from the hatchery stocks. Therefore, selective breeding has caused a significant loss of genetic variation, allelic diversity and overall heterozygosity compared to the parental generation.

The pattern of genetic relatedness has a direct functional relationship with the Ne of the population [109]. The next generation would have a higher probability of sharing the same parents if interbreeding was performed between individuals from populations with small Ne [112]. As a result, the mean and variance in pairwise relatedness within the next generation are expected to increase with decreasing Ne [109]. Therefore, it is also worth noting that extra caution should be taken when selecting broodfish of this *Tor* spp. collection for cross breeding in the future to avoid a rapid increase in genetic relatedness and reduction in Ne.

### 4.5. Population Bottleneck, Effective Population Size (Ne) and Population Assignment

In the present study, a recent population bottleneck was detected in FFRC, GPRK, HLKW, KENS, MSJ and PHG populations and a mode-shift in allele frequencies in AGHR population. Sampling error in GPRK and small sample size in AGHR and EMS populations have resulted in negative effective population size (Ne) estimates in these populations. Ne measures the rate of inbreeding and genetic drift in the population [113]. Population bottleneck and Wahlund effect could influence the Ne [114]. Generally, a mass reduction in the Ne can lead to a large decrease in SSR variations [115]. Consequently, the genetic differentiation, gene flow and genetic diversity of the population will be affected [114].

The accuracy of the population assignment test did not seem to affect much by null alleles in the present study. In the population assignment test, a high percentage (i.e., 90%) of individuals correctly assigned to respective populations were observed for the *Tor* spp. collection. The percentage has doubled the previous study (i.e., 42.8%) by [33]. As reported in many studies, SSR loci with null alleles could lower the power to correctly assign individuals in the population assignment test [100]. Therefore, loci less prone to null alleles should always be preferred in population genetic studies [93]. Thus, the population assignment test outcome is more affected by the population differentiation and might improve by having an ample number of loci [100].

### 4.6. Genetic Information and Broodstocks Management

An appropriate base population containing selected fish with desirable characteristics that harbour adequate genetic diversity is a prerequisite for successful broodstock development and effective genetic management. Therefore, the genetic information obtained from this study is essential to formulate appropriate strategies for genetic resource protection of *Tor* spp. and for their utilization in aquaculture development, especially for selective breeding programmes. The high percentages of departures from HWE as the consequences of excessive homozygosities among the SSR loci showed an urgent need for proper management strategies of these *Tor* stocks. It was evident from the genetic structures obtained that the *Tor* spp. collected for the establishment of the hatchery population comprised the natural gene pool of four distinct genetic sources. Understanding the connectivity among the populations provides a useful tool to determine appropriate strategies for fisheries conservation, effective management and genetic improvement of the Malaysian mahseer.

Analysis of genetic relationships is an essential component in a genetic improvement programme. It provides information about genetic diversity, and it also offers the platform for the stratified sampling of breeding populations [116,117,118]. For sustainable aquaculture development, strategies to minimise the loss of genetic variation of the captive breeding populations should be undertaken through minimising genetic drift, while maximising the Ne [112]. A genetic admixture of several different genetic stocks that can help increase the mean number of alleles and heterozygosity is the preferred strategy. This management strategy has been applied successfully in some aquaculture species [30,119,120,121].

Proper knowledge of stock structure is necessary to preserve genetic diversity and ensure sustainable exploitation of the broodstocks. With reasonable variability, which ranged from intermediate to high levels in the current *Tor* spp. collection, it should serve as a valuable germplasm resource and a suitable base population to start with for future utilization and genetic improvements of this species. Excessive homozygosity caused the departures from HWE we observed, highlighting the need for better management and planned breeding programmes of these *Tor* stocks.

For better prospects, *Tor* spp. stock from east Malaysia (Sabah and Sarawak) and northern Peninsular Malaysia (Kelantan) shall be included in future studies to best characterize the *Tor* spp. in Malaysia, which could better understand the current genetic status of Malaysian mahseer in the whole country. Future samples shall be obtained from more reliable sources for the stocks of Hulu Langat River and Mersing Johor populations. In genetic conservation programmes, milt samples of the *Tor* stocks from the GPRK, PHG and east Malaysia populations should be prioritized for sperm cryo-banking. Besides that, the polymorphic SSR loci with considerable genetic variations used in this study and those with private alleles are potentially useful for pedigree and parentage analyses of the new breeds from the stocks, as well as in the development of marker-assisted selection technology (MAS) for *Tor* spp. in this region. The SSR markers used in the study are expected to be useful for the ongoing inter-population diallel cross-breeding and growth performance assessments of the fingerlings produced from the same pool of candidate broodstocks. These SSR markers are also of potential use in monitoring the genetic impacts of restocking activities on the wild populations of *Tor* spp. The levels of genetic variation, which included measures of allelic diversity, overall heterozygosity, Ne and genetic relatedness, should be monitored continuously for the breeds resulting from these broodstocks.

## 5. Conclusions

The SSR genotyping in this study has successfully established the SSR profile for the *Tor* spp. collection obtained from 11 populations using 22 SSR loci. The information on SSR polymorphism and diversity, gene flow, population differentiation, genetic structure, genetic relatedness and their demographic aspects of the *Tor* spp. collection is obtained from the study. This finding facilitated the reliable classification of the *Tor* spp. stocks and provided excellent information on genetic variabilities and population genetic structures of the Malaysian mahseer stocks obtained from various geographical locations. The *Tor* spp. collection still retained their genetic variation but exhibited excessive homozygosity among individuals within population and little genetic variation transfer between the populations. Generally, the levels of genetic variation and the population structures corresponded to the geographical origins of the *Tor* spp. The private alleles we found to be present in the different populations could serve as specific markers for the respective populations. The results on the genetic diversity, genetic structure and relationships, and the methodology used in the study may be utilized in the future for constructing genetic linkage maps for marker-assisted breeding and for identifying growth trait-associated markers in *Tor* spp.

## Figures and Tables

**Table 1:** The Malaysian mahseer samples used for microsatellite genotyping in this study.

No.	Sample Population	Population ID	Origin	Year of Collection	Sample Type	Number of Samples
1	Fisheries Research Institute Glami Lemi stock	FFRC	Kenyir Lake, Terengganu	2000–2004	Frozen milt	11
2	Kg Esok, Jelebu, Negeri Sembilan	KENS	Kenaboi River, Jelebu, Negeri Sembilan	2007–2008	Frozen milt	41
3	Aquaculture Extension Center, Perlok, Jerantut, Pahang	PPAP	Pahang River	2006–2008	Frozen milt	13
4	AgroHarvest, Raub, Pahang	AGHR	Keniam River, Taman Negara	2007–2008	Frozen milt	5
5	Kelah World, Hulu Langat, Selangor ^a^	HLKW	Imported from Sumatera, Indonesia	2007–2008	Frozen milt	21
6	Grik, Perak ^b^	GPRK	Kejar Banding River, Perak	2010–2011	Scale	16
7	Raub, Pahang ^b^	PHG	Jerai River, Pahang	2016	Scale	11
8	Terengganu ^b^	TGN	Berang River, Terengganu	2016	Scale	14
9	Mersing, Johor ^c^	MSJ	Endau Rompin, Johor	2016–2017	Scale	28
10	Hulu Langat, Selangor ^c^	HLS	Hulu Langat River, Selangor	2017	Scale	20
11	Empurau, Sarawak ^c,d^	EMS	Sarawak	2017	Scale	1
					Total	181

^a^ This is a different *Tor* species from the stocks in Malaysia and morphologically most likely *T. tambroides*. The stocks from other localities were *T. tambra*; ^b^ The live broodstocks of the GPRK, TGN, and PHG populations were collected from the wild at the juvenile stage and were domesticated in the ponds until matured; ^c^ The stocks of the HLS, MSJ, and EMS populations were obtained from local fish traders at the respective localities; ^d^ A total of five fish were obtained from a local fish trader at Penang who claimed the stock was originated from Sarawak; however, only one fish survived after the quarantine period; FFRC: FRIGL stock (collected from FFRC Batu Berendam, Melaka); KENS: Kg Esok, Jelebu, Negeri Sembilan; PPAP: Aquaculture Extension Center, Perlok, Jerantut, Pahang; HLKW: Kelah World, Hulu Langat, Selangor; AGHR: AgroHarvest, Raub, Pahang; HLS: Hulu Langat, Selangor; GPRK: Grik, Perak; PHG: Raub, Pahang; MSJ: Mersing, Johor; TGN: Terengganu; EMS: Empurau, Sarawak.

**Table 2:** Microsatellite diversity and polymorphism of *Tor* spp. stocks by population.

Population ID	A_r_	MAF	A_e_	N_G_	A_p_	% Polymorphic Loci	He	Ho	Fis	PIC	HWE *p*-Value
FFRC	3.5000	0.6446	2.595	3.9545	2	77.27%	0.4093	0.4008	0.013	0.3930	0.4304
KENS	4.0909	0.6896	2.459	6.2273	3	77.27%	0.3946	0.4279	−0.089	0.3623	0.2846
PPAP	4.0909	0.6486	2.311	4.6818	11	95.45%	0.4315	0.4161	0.192	0.4144	0.2579
HLKW	6.6818	0.5097	2.145	7.8182	52	95.45%	0.5970	0.4545	0.234	0.5711	0.0740
AGHR	2.6818	0.7091	3.792	2.2273	0	77.27%	0.3264	0.3909	−0.083	0.3161	0.5232
HLS	4.2727	0.6966	2.340	4.7273	0	77.27%	0.3754	0.3273	0.142	0.3513	0.2455
GPRK	3.8636	0.6591	2.948	4.7273	3	77.27%	0.4081	0.4176	−0.003	0.3831	0.3141
PHG	2.7273	0.6736	2.226	3.1364	2	63.64%	0.3676	0.4504	−0.203	0.3395	0.3443
MSJ	5.0000	0.6412	2.729	6.1818	3	77.27%	0.4282	0.3458	0.145	0.3994	0.1805
TGN	4.6818	0.6234	3.160	5.0000	9	95.45%	0.4506	0.4513	0.023	0.4354	0.3796
EMS	1.2727	0.8636	1.273	1.0000	2	27.27%	0.0682	0.2727	−1.000	0.1023	1.0000
Mean	3.8967	0.6495	2.543	4.8682		76.45%	0.4189	0.4083	0.015	0.3966	0.3667

N_A_—total allele number, A_r_—allelic richness, MAF—major allele frequency, A_e_—number of effective alleles, N_G_—number of genotypes, A_p_—private alleles, He—expected heterozygosity, Ho—observed heterozygosity, F_IS_—inbreeding coefficient, PIC—polymorphism information content, HWE *p*-value—Exact test for HWE using a Markov chain for all loci. FFRC: FRIGL stock (collected from FFRC Batu Berendam, Melaka); KENS: Kg Esok, Jelebu, Negeri Sembilan; PPAP: Aquaculture Extension Center, Perlok, Jerantut, Pahang; HLKW: Kelah World, Hulu Langat, Selangor; AGHR: AgroHarvest, Raub, Pahang; HLS: Hulu Langat, Selangor; GPRK: Grik, Perak; PHG: Raub, Pahang; MSJ: Mersing, Johor; TGN: Terengganu; EMS: Empurau, Sarawak.

**Table 3:** Microsatellite diversity and polymorphism by locus with respective optimum Tm and product size range.

SSR Marker	Tm (°C)	Product Size (bp)	MAF	N_A_	N_G_	No. of Allele per Genotype	A_r_	He	Ho	PIC	*f*	HWE *p*-Value	Nm
BS02	48	171–189	0.5249	5	6	0.8333	2.273	0.5531	0.8122	0.4589	−0.4639	0.0000 **	3.370
BS03	50	208–224	0.7376	6	5	1.2000	2.545	0.4248	0.5193	0.3924	−0.2174	0.0001 **	1.398
BS04	65	140–174	0.7901	15	29	0.5172	3.091	0.3697	0.1713	0.3656	0.5406	0.0000 **	0.709
BS05	50	241–259	0.9448	6	9	0.6667	1.455	0.1060	0.0442	0.1046	0.5865	0.0000 **	0.805
BS06	52	204–268	0.9392	6	6	1.0000	1.727	0.1160	0.1215	0.1135	−0.0427	0.7843	1.348
BS07	50	154–232	0.3812	9	11	0.8182	3.364	0.6692	0.8287	0.6039	−0.2331	0.0000 **	1.363
BS08	50	241–257	0.5746	4	5	0.8000	2.364	0.5063	0.8232	0.3990	−0.6226	0.0000 **	6.433
BS09	51	243–259	0.9061	6	7	0.8571	2.000	0.1750	0.1768	0.1697	−0.0046	0.7765	3.096
NY01	55	223–243	0.4751	8	16	0.5000	3.364	0.6839	0.3702	0.6430	0.4631	0.0000 **	0.545
NY02	56	238–274	0.8867	12	14	0.8571	2.455	0.2107	0.1271	0.2082	0.4017	0.0000 **	0.749
NY03	66	93–105	0.5580	5	13	0.3846	3.455	0.6168	0.2265	0.5749	0.6361	0.0000 **	0.941
NY04	55	239–275	0.5608	15	39	0.3846	4.727	0.6562	0.3481	0.6416	0.4739	0.0000 **	0.878
NY05	60	168–208	0.4144	13	21	0.6190	4.545	0.7127	0.3370	0.6728	0.5311	0.0000 **	1.104
NY06	60	131–173	0.2320	23	57	0.4035	7.273	0.8783	0.8066	0.8706	0.0872	0.0000 **	0.975
NY07	55	233–245	0.9917	3	3	1.0000	1.182	0.0164	0.0055	0.0164	0.6660	0.0011 *	4.192
NY08	66	173–224	0.1409	26	71	0.3662	8.364	0.9197	0.5304	0.9185	0.4278	0.0000 **	1.095
NY09	58	239–249	0.8122	8	8	1.0000	2.636	0.3301	0.2486	0.3191	0.2521	0.0000 **	0.635
NY10	66	174–202	0.1188	26	81	0.3210	9.818	0.9238	0.8564	0.9218	0.0785	0.0000 **	1.830
NY11	60	201–283	0.1851	33	84	0.3929	10.091	0.9185	0.6630	0.9171	0.2833	0.0000 **	1.071
NY12	65	151–173	0.2293	11	34	0.3235	4.727	0.8437	0.4751	0.8296	0.4413	0.0000 **	0.486
NY13	64	150–162	0.9227	4	4	1.0000	1.364	0.1435	0.0110	0.1367	0.9238	0.0000 **	0.373
NY14	65	183–197	0.4254	6	10	0.6000	2.727	0.6597	0.4088	0.5944	0.3850	0.0000 **	0.671
		Mean	0.5796	11.36	24.23	0.6748	3.888	0.5197	0.4051	0.4942	0.2259		1.548

* *p* < 0.01 and ** *p* < 0.001; SSR—Simple sequence repeat or microsatellite, Tm—annealing temperature, MAF—major allele frequency, N_A_—number of alleles, N_G_—number of genotypes, A_r_—number of alleles per genotype, allelic richness, He—genetic diversity, Ho—observed heterozygosity, PIC—polymorphism information content, *f*—inbreeding coefficient, HWE—Hardy–Weinberg Equilibrium, Nm—estimates of gene flow. The probability of deviation from HWE was based on the Markov Chain Monte Carlo (MCMC) method.

**Table 4:** Analysis of molecular variance (AMOVA) showing the population genetic structure of the *Tor* spp. collection.

Source of Variation	d.f.	Sum of Squares	Variance Components	Percentage of Variation	*p*-Value
Among populations	10	330.674	0.87317 V_a_	14.92	*p* < 0.001
Among individuals within populations	170	936.019	0.52648 V_b_	9.00	*p* < 0.001
Within individuals	181	806.000	4.96302 V_c_	76.09	*p* < 0.001
Total	361	2072.693	5.85339	100.00	

**Table 5:** Genetic relatedness (R_xy_) among individuals within each population and their correlation coefficients based on individuals’ microsatellite (SSR) genotype data.

Population	Moment Estimators *					Likelihood Estimators *		Correlation Coefficients
	Wang (2002)	Lynch (1988) and Li et al. (1993)	Lynch and Ritland (1999)	Ritland (1996)	Queller and Goodnight (1989)	Wang (2007)	Milligan (2003)	
AGHR	−0.199 (0.030)	−0.181 (0.035)	−0.250 (0.010)	−0.223 (0.025)	−0.249 (0.037)	0.021 (0.001)	0.027 (0.002)	0.523–0.968
FFRC	−0.142 (0.061)	−0.105 (0.058)	−0.100 (0.038)	−0.100 (0.036)	−0.098 (0.052)	0.056 (0.023)	0.065 (0.026)	0.749–0.994
GPRK	−0.058 (0.026)	−0.025 (0.028)	−0.067 (0.011)	−0.066 (0.017)	−0.066 (0.029)	0.067 (0.008)	0.081 (0.010)	0.554–0.976
HLKW	−0.131 (0.075)	−0.160 (0.144)	−0.050 (0.023)	−0.057 (0.059)	−0.049 (0.126)	0.144 (0.042)	0.155 (0.045)	0.850–0.995
HLS	−0.071 (0.145)	−0.127 (0.211)	−0.053 (0.050)	−0.055 (0.037)	−0.031 (0.164)	0.191 (0.073)	0.209 (0.081)	0.820–0.996
MSJ	−0.108 (0.057)	−0.095 (0.106)	−0.037 (0.025)	−0.039 (0.040)	−0.034 (0.092)	0.144 (0.042)	0.168 (0.050)	0.817–0.990
KENS	0.024 (0.056)	0.053 (0.045)	−0.025 (0.020)	−0.024 (0.016)	−0.022 (0.042)	0.091 (0.016)	0.113 (0.020)	0.562–0.976
PPAP	−0.079 (0.222)	−0.111 (0.399)	−0.083 (0.035)	−0.102 (0.076)	−0.009 (0.100)	0.087 (0.021)	0.104 (0.025)	0.455–0.984
PHG	0.010 (0.083)	0.039 (0.065)	−0.100 (0.050)	−0.081 (0.026)	−0.097 (0.066)	0.120 (0.025)	0.127 (0.027)	0.714–0.993
TGN	−0.074 (0.048)	−0.061 (0.061)	−0.077 (0.016)	−0.080 (0.019)	−0.069 (0.045)	0.066 (0.012)	0.080 (0.014)	0.639–0.984
Overall	−0.048 (0.040)	−0.065 (0.121)	−0.006 (0.014)	−0.007 (0.020)	0.013 (0.055)	0.107 (0.026)	0.130 (0.031)	0.531–0.986

Results are presented as mean and variance (in parenthesis) values.; * The bootstrap analysis revealed no statistical differences at 0.05 between populations in mean relatedness coefficients (Rxy) for all estimators. AGHR: AgroHarvest, Raub, Pahang; EMS: Empurau, Sarawak; FFRC: FRIGL stock (collected during FFRCBatu Berendam, Melaka); GPRK: Grik, Perak; HLKW: Kelah World, Hulu Langat, Selangor; HLS: Hulu Langat, Selangor; KENS: Kg Esok, Jelebu, Negeri Sembilan; MSJ: Mersing, Johor; PHG: Raub, Pahang; PPAP: Aquaculture Extension Center, Perlok, Jerantut, Pahang; TGN: Terengganu.

**Table 6:** Bottleneck analysis within populations of *Tor* spp. studied using the Wilcoxon sign-rank test.

Populations	IAM	TPM	SMM	Mode Shift
AGHR	0.3389	0.4816	0.5912	Y
FFRC	0.0101 *	0.0797	0.3560	N
GPRK	0.0075 *	0.1123	0.5000	N
HLKW	0.0021 *	0.5407	0.9790	N
HLS	0.4633	0.9681	0.9977	N
KENS	0.0198 *	0.5550	0.9716	N
MSJ	0.0224 *	0.5367	0.8966	N
PHG	0.0067 *	0.0067 *	0.0067 *	N
PPAP	0.6586	0.9677	0.9903	N
TGN	0.1602	0.7416	0.9484	N

AGHR: AgroHarvest, Raub, Pahang; EMS: Empurau, Sarawak; FFRC: FRIGL stock (collected from FFRC Batu Berendam, Melaka); GPRK: Grik, Perak; HLKW: Kelah World, Hulu Langat, Selangor; HLS: Hulu Langat, Selangor; KENS: Kg Esok, Jelebu, Negeri Sembilan; MSJ: Mersing, Johor; PHG: Raub, Pahang; PPAP: Aquaculture Extension Center, Perlok, Jerantut, Pahang; TGN: Terengganu; IAM: infinite allele model; TPM: two-phase model; SMM: stepwise mutational model. For TPM analyses, the variance parameter was set at 30 and percent of mutations at 70% adhered to a strict stepwise mutational model. Y: yes, N: no. * Results were significant at *p* < 0.05.

**Table 7:** Population assignment of *Tor* spp. and respective estimates of effective population size (Ne) within each population.

Population	N	Ne	95% Confidence Intervals (CI)		Self-Population	Mismatched	
			Lower Bound	Upper Bound		Number (Percentage)	Population Assigned
AGHR	5	−6.1	−8.9	Infinite	5	0	
EMS	1	−0.3	−0.3	Infinite	1	0	
FFRC	11	20.9	11.2	63.8	11	0	
GPRK	16	−430.0	54.0	Infinite	16	0	
HLKW	20.9	14.2	11.1	18.7	17	4 (23.5%)	KENS(2), GPRK (2)
HLS	20	9.9	7.1	13.9	16	4 (25%)	MSJ(2), GPRK (2)
KENS	41	63.6	27.3	1162.5	41	0	
MSJ	27.9	19.6	14.2	28.3	21	7 (33.3%)	HLS(3), TGN(3), AGHR(1)
PHG	11	13.8	5.2	105.7	11	0	
PPAP	13	2.3	1.9	2.8	13	0	
TGN	14	23.1	15.5	40.0	11	3 (27.3%)	FFRC(2), AGHR(1)
Total (%)	181				163 (90%)	18 (10%)	

AGHR: AgroHarvest, Raub, Pahang; EMS: Empurau, Sarawak; FFRC: FRIGL stock (collected from FFRC Batu Berendam, Melaka); GPRK: Grik, Perak; HLKW: Kelah World, Hulu Langat, Selangor; HLS: Hulu Langat, Selangor; KENS: Kg Esok, Jelebu, Negeri Sembilan; MSJ: Mersing, Johor; PHG: Raub, Pahang; PPAP: Aquaculture Extension Center, Perlok, Jerantut, Pahang; TGN: Terengganu; N—sample size. The Ne analysis included alleles with frequencies greater than 0.01, with the upper and lower 95% confidence intervals.

## Data Availability

Data sharing not applicable.

## Supplementary Materials

The following are available online at https://www.mdpi.com/article/10.3390/ani11092633/s1, Figure S1: Total genomic DNA extracted from some of the (a) post-thawed milt samples (i.e., sample 1–12) and (b) scale samples (i.e., sample 13–28) of *Tor* spp. after electrophoresis on 1% agarose gel in 1× TAE buffer. λ is the lambda Hind III DNA marker. Figure S2: PCR products amplified for marker NY02 from total genomic DNA extracted from (a) milt sample and (b) scale sample of *Tor* spp. after electrophoresis on 1.8% agarose gel has yielded targeted fragments at the sizes between 238–274 bp. 100 bp is the GeneRuler DNA ladders. Figure S3: PCoA plot based on genetic distance matrix of 181 *Tor* spp. collected from eleven populations utilizing data from 22 SSR genotype with data standardization. Figure S4: Magnitude of delta K (ΔK) statistics for the *Tor* spp. collection based on 22 microsatellite loci. ΔK as a function of the number of putative genetic clusters, K. The most likely K value identified, i.e., with the highest value was K = 4. Table S1: List of microsatellites (simple sequence repeat, SSR) markers loci and primer sequences used in genotyping of *Tor* spp. Table S2: Microsatellite diversity and polymorphism of *Tor* spp. by different sample types. Table S3: Summary of causes attributed to the departure from HWE in each of the Tor populations. Table S4: Pairwise Nei’s genetic distance coefficient (below diagonal) and pairwise FST values (above diagonal) for the *Tor* spp. populations studied.

## Appendix A

**Table A1:** Summary of private alleles (A_p_), null alleles (N_0_), and the occurrences of stuttering (S) in each *Tor* spp. population and respective SSR markers involved.

SSR ID	Populations																																
	AGHR			FFRC			GPRK			HLKW			HLS			KENS			MSJ			PHG			PPAP			TGN			EMS		
	A_p_	N_0_	S	A_p_	N_0_	S	A_p_	N_0_	S	A_p_	N_0_	S	A_p_	N_0_	S	A_p_	N_0_	S	A_p_	N_0_	S	A_p_	N_0_	S	A_p_	N_0_	S	A_p_	N_0_	S	A_p_	N_0_	S
BS02	-	-	-	-	-	-	-	-	-	173, 185	√	√	-	-	-	-	-	-	-	-	-	-	-	-	189	-	-	-	-	-	-	-	-
BS03	-	-	-	-	-	-	-	-	-	-	-	√	-	-	-	-	-	-	-	-	-	-	-	-	448, 450	-	-	-	-	-	-	-	-
BS04	-	-	-	-	-	-	-	-	-	148, 150, 152, 154, 156, 160	√	√	-	√	-	-	-	-	-	√	-	-	-	-	140	-	-	-	-	-	-	-	-
BS05	-	-	-	-	-	-	-	-	-	241, 257	√	√	-	-	-	-	-	-	-	-	-	-	-	-	259	-	-	-	-	-	-	-	-
BS06	-	-	-	232	-	-	-	-	-	254	-	-	-	-	-	-	-	-	-	-	-	-	-	-	252	-	-	256	-	-	-	-	-
BS07	-	-	-	-	-	-	-	-	-	170	-	-	-	-	-	154, 156	-	-	-	-	-	-	-	-	232	-	-	-	-	-	-	-	-
BS08	-	-	-	-	-	-	-	-	-	-	-	-	-	-	-	-	-	-	-	-	-	-	-	-	-	-	-	241	-	-	-	-	-
BS09	-	-	-	-	-	-	-	-	-	-	-	-	-	-	-	249	-	-	-	-	-	-	-	-	-	-	-	-	-	-	-	-	-
NY01	-	-	-	-	-	-	-	-	-	233, 241, 243	√	-	-	√	√	-	-	-	-	√	√	-	-	-	-	-	-	-	-	-	-	-	-
NY02	-	-	-	-	-	-	-	-	-	238, 250, 252, 254, 256, 258, 262, 266, 274	√	-	-	-	-	-	-	-	-	-	-	-	-	-	-	-	-	-	-	-	-	-	-
NY03	-	-	-	-	√	√	-	√	-	-	√	-	-	√	-	-	√	√	-	√	√	-	-	-	-	√	-	-	-	-	-	-	-
NY04	-	-	-	-	√	-	239	-		269	√	-	-	√	-	-	√	√	-	√	-	243	-	-	-	-	-	275	√	-	-	-	-
NY05	-	-	-	-	-	-	-	√	√	168, 186, 188, 196, 200, 208	√	-	-	√	√	-	√	√	-	√	√	-	√	-	-	√	√	-	√	√	-	-	-
NY06	-	-	-	168	-	-	-	-	-	130, 132, 136, 160, 162	-	-	-	√	-	-	-	-	-	√	-	-	-	-	-	-	-	176	-	-	-	-	-
NY07	-	-	-	-	-	-	-	-	-	-	-	-	-	-	-	-	-	-	-	-	-	-	-	-	233	-	-	241	-	-	-	-	-
NY08	-	-	-	-	√		-	√	√	173, 183, 187	√	-	-	√	-	-	√	√	225	√	-	195	-	-	193	√	-	229	√	-	177	-	-
NY09	-	-	-	-	-	-	-	-	-	243, 249, 255	√	√	-	-	-	-	-	-	-	-	-	-	-	-	239	-	-	-	-	-	-	-	-
NY10	-	-	-	-	-	-	-	-	-	182	-	-	-	-	-	-	-	-	222	√	-	-	-	-	-	-	-	188		-	178	-	-
NY11	-	-	-	-	√	-	212	-	-	208, 218, 220, 254, 270, 282	√	-	-	√	-	-	-	-	262	√	-	-	-	-	-	-	-	230, 256	√	-	-	-	-
NY12	-	-	-	-	-	-	-	-	-	155	√	-	-	√	√	-	-	-	-	√	√	-	-	-	-	-	-	-	√	-	-	-	-
NY13	-	-	-	-	-	-	-	-	-	162	√	√	-	-	-	-	-	-	-	-	-	-	-	-	150	-	-	-	-	-	-	-	-
NY14	-	-	-	-	-	-	197	-	-	195	√	√	-	-	-	-	-	-	-	-	-	-	-	-	-	-	-	-	-	-	-	-	-
Total	0	0	0	2	4	1	3	3	2	52	14	7	0	9	3	3	4	4	3	10	4	2	1	0	11	3	1	9	5	1	2	0	0

√ Yes; - No.
