# Supplementary material for: Microsatellite Characterization of Malaysian Mahseer (Tor spp.) for Improvement of Broodstock Management and Utilization
Source: Animals (Basel). 2021 Sep 8;11(9):2633. doi: 10.3390/ani11092633 (PMC8471032; doi:10.3390/ani11092633)
Supplement: Supplementary file 1 [file animals-11-02633-s001.zip › animals-1220926 Supplementary Material.pdf]

Supplementary Materials

Table S1. List of microsatellites (simple sequence repeat, SSR) markers loci and primer sequences used in genotyping of *Tor* spp.

No	SSR ID	Locus (Genbank accession number)	Repetitive Motif	Primer Sequences 5′ – 3′	Dye colour	Optimum Dilution Factor
1	NY01	Tt1.A06 (DQ778018)	$(ATTT)_3(GTTT)_4\,(ATT)_8$	F: CCGAAATGCATTCTTGTCTT<br>R: GGACTGACACTGGGGATCAT	HEX	3000
2	NY02	Tt1.B01 (DQ778019)	$(AC)_8AT(AC)_3$~$(TC)_3$	F: GAGGGGCATTTTGTTCTTGA<br>R: GCTTCCCCTCATAAGCCTTC	6FAM	3000
3	NY03	Tt1.B08 (DQ778020)	$(TG)_{10}$	F: GCAGAACAACATTATGCCTATGA<br>R: CTCAATGATTCACTGCAGCAC	6FAM	100
4	NY04	Tt1.C06 (DQ778021)	$(AC)_{12}$~$(AC)_3$~ $(AC)_3$~$(AC)_4$	F: GCCCGTGCAGACCTCTAA<br>R: TTGGGAAAAATGCTTCATAAAA	HEX	100
5	NY05	Tt1.C10 (DQ778022)	$(TG)_{13}$	F: GCTGAAGCAGGTGAATCTGA<br>R: TGATGCCTGTCAAACCTGTG	HEX	1000
6	NY06	Tt1.E11 (DQ778025)	$(TG)_{23}$	F: GAGTCCCTACAGACGTATTTCCA<br>R: TTCAGCTCAGAGGGGACACT	6FAM	100
7	NY07	Tt1.F02 (DQ778026)	$(TG)_2TA(TG)_4TC(TG)_2$	F: CATGGACCAAATTACAAGGATTT<br>R: AACCTGTGAGGGATGTCCAG	TAMRA	3000
8	NY08	Tt2.B02 (DQ778028)	$(TG)_{15}$~$(TG)_3$	F: CTGGGAACGTCAGTTTACGG<br>R: GTCCCCACAAGGATAGCAGA	ROX	100
9	NY09	Tt2.B07 (DQ778029)	$(TG)_3(TC)_6TT(TC)_3TT(TC)_6$	F: TGGAAATTGAGACAAAGCTTCA<br>R: TATGTGGTTTCAGGCAGCAG	ROX	2000
10	NY10	Tt2.B10 (DQ778030)	$(AC)_{15}$	F: CTGGGAACGTCAGTTTACGG<br>R: GTCCCCACAAGGATAGCAGA	ROX	100
11	NY11	Tt2.D01 (DQ778031)	$(TG)_{15}$	F: CCATTACGCCTTTGGAGTGT<br>R: TGGGAGATGTTGTTTCTCCA	HEX	500
12	NY12	Tt2.F04 (DQ778032)	$(AC)_{11}$	F: ATGCCAGCTACAGGTCCAAT<br>R: CGTGTGTATGATGCCACCTC	TAMRA	1000
13	NY13	Tt2.F07 (DQ778033)	$(AG)_9$	F: GAGACGACTCTAGTCGCTGACA<br>R: GTGTGGCCAGTGTAGCTGAA	6FAM	3000
14	NY14	Tt2.H08 (DQ778034)	$(TC)_{14}$	F: GGCTGTGAATGTGTTTGTGG<br>R: GCCAGGATGATGAGCATGTA	TAMRA	500
15	BS01*	SKVJ1-16 (EF472553.1)	$(TG)_9$~$(TG)_3$~$(TG)_3$	F: CAAGAAGACAACAAGGAACTGC<br>R: TGTTTCTCGAGGGGACTACT	6-FAM	NA
16	BS02	SKVJ1-12 (EF472549.1)	$(AC)_{10}$~$(TCC)_3$~ $(TT)_3$~$(CT)_3$	F: CTCTTGCTTCTCGAGGGACT<br>R: TCACTAACAACTCCACCGCA	6-FAM	100
17	BS03	SKVJ1-11 (EF472548.1)	$(CT)_6$~$(TC)_3$~$(C\ T)_3$	F: CTCTTGCTTCTCGAGGGACT<br>R: AAAGGCAATCGGAATGCAGC	HEX	2000
18	BS04	SKVJ1-9 (EF472546.1)	$(CA)_8$~$(TA)_4$	F: CCAAACTGTCCATGCTGTCC<br>R: GTTGGCTCTCCGTTCTTCAG	TAMRA	3000
19	BS05	SKVJ1-8 (EF472545.1)	$(AC)_8$~$(CA)_{17}$~ $(CA)_3$	F: CTCTTGCTTCTCGAGGGAC<br>R: AGCCATGAATCAAAGACGCC	ROX	3000
20	BS06	SKVJ1-6 (EF472543.1)	$(AG)_3$~$(CT)_4$~$(AG)_6$	F: CCTGCCATGCACAGAAAACC<br>R: CTTCTCGAGGGACTATGAGCG	HEX	500
21	BS07	SKVJ1-5 (EF472542.1)	$(AC)_3$~$(AC)_6$~$(AC)_3$~$(CA)_8$	F: CTCTTGCTTCTCGAGGGAC<br>R: ACAAGAAGACAACAAGGAACTGC	TAMRA	100

22	BS08	SKVJ1-4 (EF472541.1)	$(CA)_{10}$~$(CA)_3$~$(G C)_3$	F: CTCTTGCTTCTCGAGGGAC<br>R: AATCCAGTCATTGTGAGCGC	ROX	1000
23	BS09	SKVJ1-2 (EF472539.1)	$(CT)_7$~$(CA)_6$~$(AC)_{13}$~$(CA)_3$~$(CA)_3$	F: TTCTCGAGGGACTATTGGGG<br>R: GCCATGAATCAAAGACGCCA	TAMRA	100
24	BS010*	SKVJ1-1 (EF472538.1)	$(CA)_3$~$(AC)_3$~$(AC)_3$~$(CA)_8$	F: CTTGCTTCTCGAGGGACT<br>R: GCACACTGAAACTAAGAGACA	ROX	NA

* Primer pair BS01 and BS10 failed to produce specific fragments and were omitted from the SSR genotyping study; NA – not applicable

Genomic DNA extraction

Genomic DNA extraction typically involved lysis, washing, and elution procedures. Briefly, the sperm pellet or scale sample was mixed well in 180 µL ATL buffer, 20 µL proteinase K, and incubated at 56 °C. The duration of incubation was 3 hrs and overnight for sperm pellet and scale samples, respectively. For thawed milt samples, an additional step of treatment with 20 µL 1M dithiothreitol (DTT) was performed during the lysis procedure. After the lysis procedure, the samples were then treated with 400 µg RNase A at room temperature for 2 min. After that, the mixture was precipitated with 200 µL AL buffer and 200 µL absolute ethanol before being subjected to centrifugation at 8,000 rpm for 1 min. The DNA pellet was subsequently washed with 500 µL AW1 buffer and centrifuged again at 8,000 rpm for 1 min. After that, the supernatant was discarded. The DNA pellet was then washed with 500 µL AW2 buffer and centrifuged at 14,000 rpm for 3 min. Finally, the DNA pellet was eluted in 150 µL AE buffer.

Quality of total genomic DNA

The concentrations of the total genomic DNA extracted from the milt and scale samples ranged between 2.7 ng/µL to 335.0 ng/µL (Figure S1). The DNA purity ranged from intermediate to high, with the A260/A280 ratio being between 1.36 to 1.83

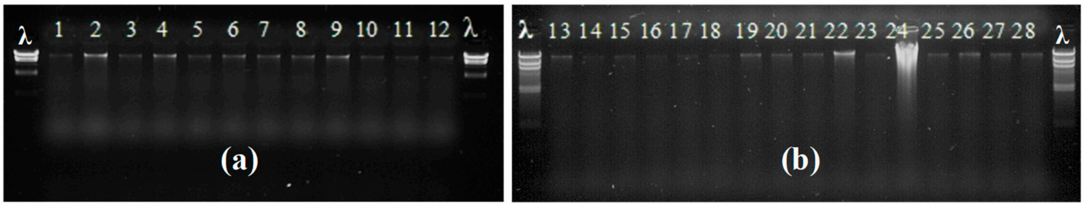

Figure S1. Total genomic DNA extracted from some of the a) post-thawed milt samples (i.e., sample 1 - 12) and b) scale samples (i.e., sample 13 - 28) of *Tor* spp. after electrophoresis on 1% agarose gel in 1x TAE buffer. λ is the lambda Hind III DNA marker.

PCR amplification and fragment analysis

Among the 24 primer pairs tested, only 22 primers (91.7%) showed successful amplification following the predefined optimum annealing temperature and yielded fragments at the targeted sizes (Table 3). Amplifications by primers BS01 and BS10 were not specific and showed a multi-locus profile, although repeated attempts were made. Therefore, these two primer pairs were not included in the SSR genotyping. The SSR profile generated from the SSR-PCR amplification using DNA extracted

from both frozen milt and scale samples showed fragments with identical sizes as targeted (Figure S2).

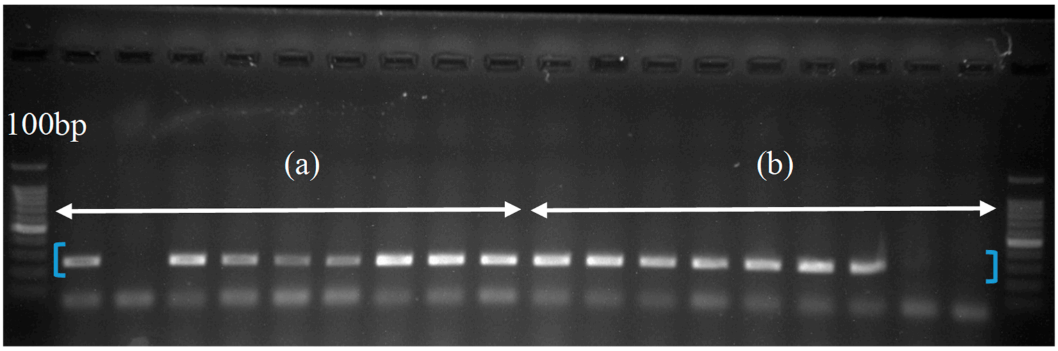

Figure S2. PCR products amplified for marker NY02 from total genomic DNA extracted from a) milt sample and b) scale sample of *Tor* spp. after electrophoresis on 1.8% agarose gel has yielded fragments at the targeted sizes ([ ]) between 238 – 274 bp. 100 bp is the GeneRuler DNA ladders.

SSR genetic diversity and polymorphism by locus

Among the 22 microsatellite loci examined across samples in all populations, all loci were polymorphic (100%) and produced a total of 250 alleles and 533 genotypes. The level of SSR genetic diversity in each locus was presented in Table 3. The total number of alleles (N_A) and genotypes (N_G) at a single locus ranged between 3-33 and between 3-84 in NY07 and NY11, respectively. The mean number of alleles per locus was 11.36, while the mean number of genotypes per locus was 24.23. Allelic richness (A_r) ranged from 1.182 (NY07) to 10.091 (NY11), with an average of 3.888. The least polymorphic marker was NY07 (3 alleles). On the other hand, the number of alleles generated per genotype ranged from 0.3210 in NY10 to 1.2000 in BS03, with the mean value of 0.6748. The MAF for each locus ranged from 0.1188 in NY10 to 0.9917 in NY07 and with a mean value of 0.5796.

Expected heterozygosity (H_e) varied from 0.0164 (NY07) to 0.9238 (NY10) with a mean value of 0.5197. Meanwhile, observed heterozygosities (H_o) ranged from 0.0055 (NY07) to 0.8564 (NY10). The mean value of H_o for all loci was 0.4051. Generally, the value of expected heterozygosity (He) was higher than the value of observed heterozygosity (Ho) in most loci, indicating the occurrence of deviations from HWE. Based on the exact test performed, it was revealed that 20 out of the 22 o SSR loci (90.9%) were significantly ($p<0.05$) deviated from HWE after sequential Bonferroni adjustment. Of all the SSR loci, only BS06 and BS09 were found conforming to HWE ($p>0.05$).

In this study, the gene flow over all loci was low, with the overall number of migrants (Nm) being 1.548. Of all loci, the highest Nm was in BS08 (6.433), while the lowest Nm was in NY13 (0.373) (Table 3). Overall, PIC was in the range of 0.0164 - 0.9218, with the mean value of 0.4942. Eleven SSR loci (50%) were found to be highly informative, with PIC value >0.5. The PIC values were reasonably informative (i.e., 0.25 < PIC< 0.5) and slightly informative (i.e., PIC < 0.25) in five (22.7%) and six (27.3%) SSR loci, respectively.

SSR genetic diversity and polymorphism by different sample types

Results of the SSR genotyping were also analyzed according to different sample types (frozen milt VS scale samples) to compare the levels of genetic diversity and polymorphism of the *Tor* spp. samples collected between the years 2000 to 2008 and 2010 to 2017.

A total of 216 alleles and 360 genotypes were generated from cryopreserved milt samples, with an average of 9.82 alleles and 16.36 genotypes per primer (Table 5). In the live broodfish collection, a total of 172 alleles and 324 genotypes were generated, with an average of 7.82 alleles and 14.73 genotypes per primer. The MAF was 0.5852 in the cryopreserved milt samples, while it was 0.6088 in the live broodstock samples. The total numbers of private alleles generated from the cryopreserved milt samples were higher (68) than from the live broodfish collection (19). The percentage of polymorphic loci for the cryopreserved milt samples (84.5%) was also higher than for the live broodstock (69.7%). In general, the cryopreserved milt samples showed higher gene diversity (0.5339), heterozygosity (0.4271), and PIC value (0.5080) than the live broodfish collection. The gene diversity, heterozygosity and PIC value for live broodfish collection were 0.4669, 0.3828 and 0.4390, respectively.

When EMS, with the lowest gene diversity and polymorphism, was excluded from the analysis, it caused slight changes. Still, it was insignificant in the overall MAF, the number of alleles, genotypes, and private alleles, He, Ho, and PIC. However, the percentage of polymorphic loci increased from 69.7% to 78.2% when EMS was excluded from the analysis. Generally, genetic diversity and polymorphism were higher for *Tor* spp. samples collected between 2000–2008 (cryopreserved milt) than those obtained between 2010–2017.

Table S2. Microsatellite diversity and polymorphism of *Tor* spp. by different sample types

Sample type	N	MAF	N_A (N_A per primer)	N_G (N_G per primer)	A_P	% Polymorphic Loci	He	Ho	PIC
Cryopreserved milt	91	0.5852	216 (9.8182)	360 (16.3636)	68	84.5%	0.5339	0.4271	0.5080
Live broodfish	90	0.6088	172 (7.8182)	324 (14.7273)	19	69.7%	0.4669	0.3828	0.4390
Live broodfish (excluded population EMS)	89	0.6085	170 (7.7273)	321 (14.5909)	17	78.2%	0.4673	0.3841	0.4394

EMS Empurau Sarawak, N = sample size. Major allele frequency (MAF), number of alleles (N_A), number of genotypes (N_G), number of private alleles (A_P), percentage of polymorphic loci, gene diversity (He), observed heterozygosity (Ho), polymorphism information content (PIC)

Table S3. Summary of causes attributed to the departure from HWE in each of the *Tor* populations.

Causes of departure from HWE	Populations										
	EMS	GPRK	PHG	KENS	PPAP	AGHR	FFRC	TGN	HLS	MSJ	HLKW
Inbreeding	X	X	X	X	√	X	√	√	√	√	√
Bottleneck	NA	√	√	√	X	X	√	X	X	√	√
Mode shift in allele frequency	NA	X	X	X	X	√	X	X	X	X	X
Null alleles and stuttering	X	√	√	√	√	X	√	√	√	√	√
Small sample size	√	X	X	X	X	√	X	X	X	X	X
Sampling error	X	√	X	X	X	X	X	X	X	X	X

EMS: Empurau, Sarawak; GPRK: Grik, Perak; PHG: Raub, Pahang; KENS: Kg Esok, Jelebu, Negeri Sembilan; PPAP: Aquaculture Extension Center, Perlok, Jerantut, Pahang; AGHR: AgroHarvest, Raub, Pahang; FFRC: FRIGL stock (collected from FFRC Batu Berendam, Melaka); TGN: Terengganu; HLS: Hulu Langat, Selangor; MSJ: Mersing, Johor; HLKW: Kelah World, Hulu Langat, Selangor

√ – Yes, X – No, NA – not applicable

Table S4. Pairwise Nei's genetic distance coefficient (below diagonal) and pairwise F_{ST} values (above diagonal) for the *Tor* spp. populations studied.

**OTU**	**AGHR**	**EMS**	**FFRC**	**GPRK**	**HLKW**	**HLS**	**KENS**	**MSJ**	**PHG**	**PPAP**	**TGN**	
**AGHR**	-	0.192	0.083*	0.187*	0.197*	0.147*	0.160*	0.091*	0.211*	0.083*	0.036	**AGHR**
**EMS**	0.332	-	0.144	0.112*	0.135*	0.161	0.235*	0.072	0.227	0.136	0.104	**EMS**
**FFRC**	0.183	0.323	-	0.191*	0.189*	0.111*	0.126*	0.089*	0.145*	0.085*	0.050*	**FFRC**
**GPRK**	0.240	0.276	0.265	-	0.194*	0.137*	0.237*	0.092*	0.210*	0.178*	0.136*	**GPRK**
**HLKW**	0.374	0.423	0.369	0.335	-	0.225*	0.235*	0.193*	0.212*	0.189*	0.188*	**HLKW**
**HLS**	0.205	0.280	0.180	0.170	0.333	-	0.160*	0.016	0.169*	0.140*	0.089*	**HLS**
**KENS**	0.202	0.353	0.190	0.287	0.342	0.178	-	0.145*	0.127*	0.090*	0.137*	**KENS**
**MSJ**	0.172	0.275	0.177	0.154	0.318	0.062	0.191	-	0.150*	0.101*	0.061*	**MSJ**
**PHG**	0.293	0.351	0.270	0.293	0.367	0.239	0.174	0.261	-	0.123*	0.126*	**PHG**
**PPAP**	0.196	0.342	0.208	0.256	0.339	0.201	0.148	0.180	0.214	-	0.074*	**PPAP**
**TGN**	0.142	0.321	0.136	0.223	0.342	0.171	0.173	0.153	0.258	0.188	-	**TGN**
	**AGHR**	**EMS**	**FFRC**	**GPRK**	**HLKW**	**HLS**	**KENS**	**MSJ**	**PHG**	**PPAP**	**TGN**	

AGHR: AgroHarvest, Raub, Pahang; EMS: Empurau, Sarawak; FFRC: FRIGL stock (collected from FFRC Batu Berendam, Melaka); GPRK: Grik, Perak; HLKW: Kelah World, Hulu Langat, Selangor; HLS: Hulu Langat, Selangor; KENS: Kg Esok, Jelebu, Negeri Sembilan; MSJ: Mersing, Johor; PHG: Raub, Pahang; PPAP: Aquaculture Extension Center, Perlok, Jerantut, Pahang; TGN: Terengganu

*Indicates a significant difference at $p < 0.05$ level.

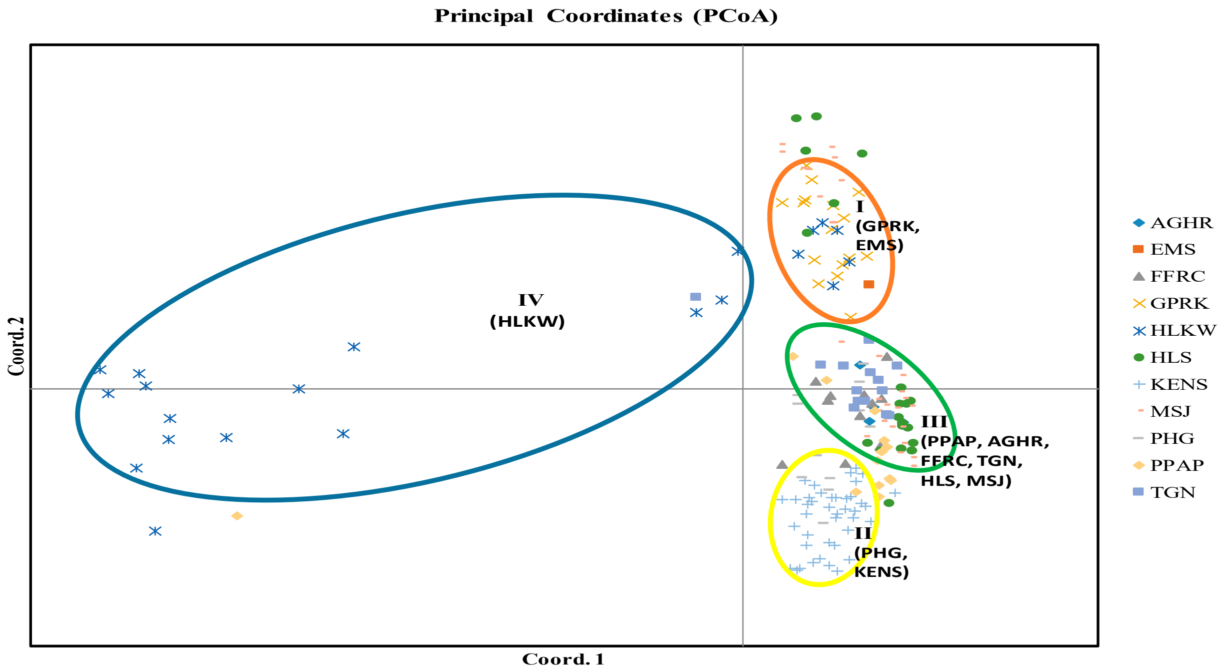

Figure S3. PCoA plot based on genetic distance matrix of 181 *Tor* spp. collected from eleven populations utilizing data from 22 SSR genotype with data standardization

HLKW: Kelah World, Hulu Langat, Selangor; EMS: Empurau, Sarawak; PHG: Raub, Pahang; GPRK: Grik, Perak; HLS: Hulu Langat, Selangor; MSJ: Mersing, Johor; KENS: Kg Esok, Jelebu, Negeri Sembilan; PPAP: Aquaculture Extension Center, Perlok, Jerantut, Pahang; AGHR: AgroHarvest, Raub, Pahang; FFRC: FRIGL stock (collected during FFRC Batu Berendam, Melaka); TGN: Terengganu

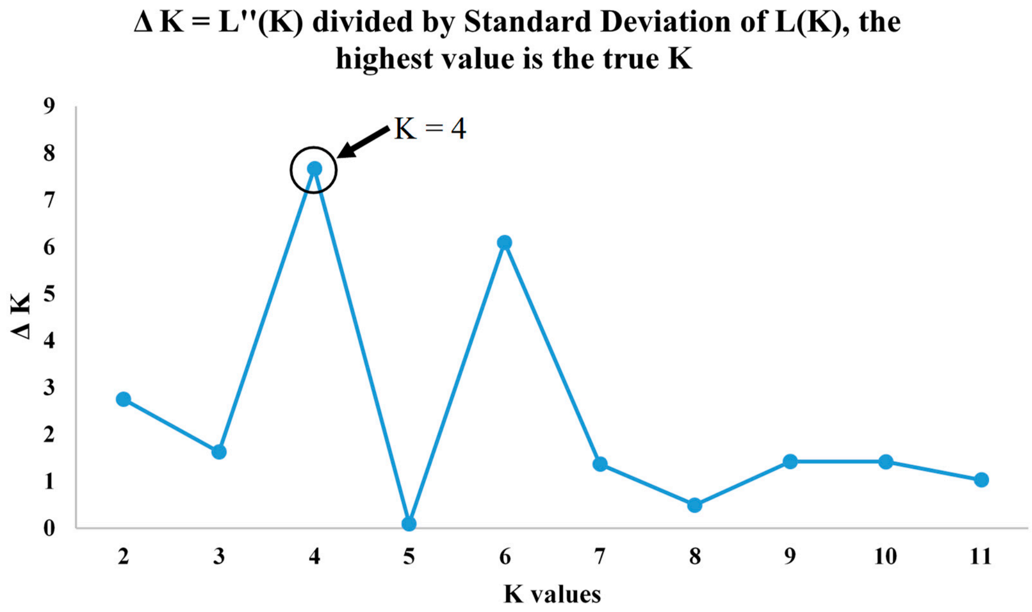

Figure S4. Magnitude of delta K (ΔK) statistics for the *Tor* spp. collection based on 22 microsatellite loci. ΔK as a function of the number of putative genetic clusters, K. The most likely K value identified, i.e., with the highest value was K =4.
